# New hit compounds against *Trypanosoma cruzi* derived from mining yeast chemogenomic profiling data

**DOI:** 10.1128/aac.00117-26

**Published:** 2026-06-04

**Authors:** Mercedes Didier Garnham, Lionel Urán Landaburu, Emir Salas Sarduy, Fernán Agüero

**Affiliations:** 1 Instituto de Investigaciones Biotecnológicas (IIBIO), Consejo Nacional de Investigaciones Científicas y Técnicas (CONICET) 62873 , San Martín, Buenos Aires, Argentina; 2 Escuela de Bio y Nanotecnologías (EByN), Universidad Nacional de San Martín (UNSAM) 28221 , San Martín, Buenos Aires, Argentina; University Children's Hospital Münster, Münster, Germany

**Keywords:** drug repositioning, chemical genomics, drug discovery, Chagas disease

## Abstract

Chagas disease, caused by *Trypanosoma cruzi*, remains a major neglected tropical disease with limited therapeutic options. To accelerate drug discovery, we developed a bioinformatic and chemoinformatic pipeline that integrates chemogenomic profiling data from *Saccharomyces cerevisiae* with orthology and essentiality information from trypanosomatids. First, we integrated data from five reference yeast chemogenomic screens that assayed a total of 2,788 compounds against a collection of 5,811 gene-deletion strains, thus providing a rich matrix of ∼270,000 gene-drug associations. Using these data as input, the pipeline filtered and prioritized 193 candidate bioactive compounds with links to conserved parasite orthologs. Following manual curation, 22 compounds were selected for experimental evaluation. *In vitro* assays against intracellular *T. cruzi* revealed two novel hits with micromolar EC${}_{50}$ values and favorable selectivity indices. This work demonstrates that comparative chemogenomics can be leveraged for drug repositioning in parasitic protozoa, providing a tractable strategy to enrich lead-like compounds, and expand the chemical space available for Chagas disease drug discovery. Importantly, both the curated yeast chemogenomic data set and the analysis pipeline are reusable resources, and the prioritized compound list offers a ready-to-test chemical space that can be further explored, validated, and expanded by other researchers and for additional organisms.

## INTRODUCTION

Trypanosomatids are a group of single-celled parasitic eukaryotes that belong to the order Kinetoplastida. These parasites typically have a complex life cycle, involving multiple developmental stages and often alternating between insect vectors and mammalian hosts, posing significant challenges for the development of effective treatments and control strategies (1). This group of parasites includes species like *Trypanosoma cruzi*, the etiological agent of Chagas disease (2, 3); *Trypanosoma brucei*, the causative agent of African trypanosomiasis (sleeping sickness) (4); and *Leishmania spp*., which are responsible for various forms of leishmaniasis, a disease complex affecting the skin, mucous membranes, and internal organs (5).

All these diseases are considered Neglected Tropical Diseases (NTDs) (6–8) and are often associated with poverty, affecting roughly 1.5 billion people, mainly in Africa, Asia, and the Americas (9), and ranking sixth in the list of leading causes of life years lost to disability and premature death (10). Historically, the lack of interest from the pharmaceutical industry, in combination with little investment in public health research programs in the affected countries, resulted in a deficiency of available drugs to control the majority of these diseases (11). Moreover, currently used drugs have well-known limitations in terms of cost, difficulties in administration, efficacy, or safety profiles (12).

The drug discovery enterprise is expensive and lengthy, currently taking 12–15 years and costing $247 million to $1.42 billion USD dollars (13, 14). In a regular drug discovery pipeline, preclinical research can take up half of that time and cost nearly 10% of those figures (15). More importantly, in the pipeline for NTDs drug discovery, only one out of five pre-clinical research lines survive long enough to make it into the subsequent clinical phases, mainly because of funding shortages (16).

A common practice to minimize both the cost and time of drug development is to explore novel applications for existing compounds (17). While drug repurposing—traditionally defined as finding new uses for approved drugs or those that failed clinical trials without showing toxicity—has been a central strategy, the scope of repositioning can be broadened. Chemogenomic screens, which assess the susceptibility or resistance of model organisms carrying genome-wide genetic modifications, are increasingly used to infer drug mechanisms of action at scale (18, 19). The data generated from these large-scale studies can be repositioned (i.e., reassessed or reanalyzed) to identify potential therapeutic relevance to neglected diseases and other non-traditional targets, expanding the concept of repurposing beyond approved drugs to include a wider array of chemical entities (20, 21).

In a less strict interpretation, repurposing of chemical leads across targets and organisms can benefit the drug discovery pipeline of NTDs by leveraging research programs focused on other diseases (22–24). One such example is the use of antifungal compounds against *T. cruzi*, which has a similar sterol biosynthetic metabolism (25). Furthermore, many antifungals showed trypanosomal or leishmanicidal activity (26, 27) and were tested in clinical trials (28–32).

While very few pharmacogenomic assays have been conducted in pathogens that cause neglected diseases (33–35), a reasonable number of these were carried out in model organisms, such as the yeast *Saccharomyces cerevisiae* (36–41) and *Candida albicans* (42). Chemogenomic assays in yeast vary in extent and genome coverage, but they all rely on measuring drug-induced haploinsufficiency—the observed growth sensitivity in the presence of a drug of a diploid yeast strain, which is hemizygous for the gene encoding the target (36). In these assays, populations of barcoded cells carrying individual allele knockouts under drug pressure are sequenced, and barcode counts provide a measure of fitness for diverse mutants. Because some genes cannot be knocked out completely without compromising cell viability, two main types of profiles can be observed: the haploinsufficient profile (HIP) that captures the growth fitness of diploid yeast cells that are hemizygous for one gene, and the homozygous profiles (HOP) that measure the growth fitness of diploid yeast with a complete gene knocked out in both chromosomes (41).

These assays have proven valuable to suggest drug-target links that were later validated *in vitro* in yeasts (43–45), along with a handful of cross-species studies conducted from *S. cerevisiae* to humans (46, 47). Recently, the data from the latest chemogenomic profiling publications have been compared for the reproducibility and accuracy of the protocol, showing robust response signatures (48). This poses a welcoming scenario for drug repurposing in NTDs.

We hereby present a data integration pipeline that allowed the mapping of yeast chemogenomic profiling data and the creation of repurposing screening libraries for *T. cruzi*. Experimental validation of a subset of compounds from these libraries led to the identification of repurposable drugs with anti-trypanosomal activity *in vitro*.

## MATERIALS AND METHODS

### Data mining and standardization

Data from each data set in Table 1 were downloaded from the respective supplementary data associated with each publication or from dedicated websites associated with these publications (49). Each data set came with their own unique formatting. A large effort was put into mining and standardizing data from different sets. In all cases, HIP profiles were transformed into a collection of gene-drug putative association arrays in the form of cross-references of ORF (target) names and a SMILES string as a descriptor of drugs and other bioactive compounds. Some criteria could be applied to all data sets, such as (i) keep only gene-drug tuples with a positive effect (e.g., observed chemical-induced haploinsufficiency); (ii) discard gene-drug tuples where either element of the array could not be mapped to the standard reference genome or public domain compound databases; and (iii) discard gene-drug tuples where the same gene had been observed to display sensitivity to a wide variety of conditions (termed MDR, multiple drug resistance, genes) to avoid noisy or promiscuous data (this parameter was set at 20%, e.g., we discarded any gene presenting sensitivity in more than 20% of the conditions tested). Other criteria used for standardization were unique for each data set.

**TABLE 1:** Data used to derive chemogenomic associations

Input data set	Assay		Resulting data set				
Reference	Drugs^*a*^	Deletion strains	Drugs^*b*^ (processed)	Drugs^*c*^ (unique)	Targets^*d*^ (processed)	Targets^*e*^ (unique)	Drug-target tuples
Giaever (37)	10	∼6,000	9	9	226	226	251
Hillenmeyer (38)	726	∼6,000	403	377	527	521	5,744
Hoepfner (40)	1,776	∼6,000	147	133	1,520	57	11,968
Lee (41)	3,250	∼1,100	341	305	156	2	437
Wildenhain (50)	5,518	242	1,964	1,820	242	7	45,871
Total				2,788		5,811	269,941

^*a*^ Total, as reported in the Reference Data set.

^*b*^ Effective, compounds processed.

^*c*^ Cumulative compounds processed.

^*d*^ Effective, genes processed.

^*e*^ Cumulative genes processed.

Once in this form, mapping each drug to the corresponding SMILES string was performed by (i) resolving the drug name to a SMILES string using the NCI Chemical Identifier Resolver through its API (51), (ii) using the PubChem PUG-REST interface to retrieve SMILES from PubChem ID (52), or (iii) directly using compound data in SMILES format supplied by the authors (in the HIP profiles or supplementary data). This semi-automated process, however, was not sufficient to process and standardize all the data, and manual curation was needed to either rename compounds to a common drug name or draw some compounds into specialized chemical applications such as Marvin Sketch (ChemAxon) to obtain canonical SMILES representations. This manual curation process was not complete; hence, the number of effectively processed compounds from each reference data set (Table 1) is often less than the number of compounds mentioned in the original publications.

For gene mapping, the *S. cerevisiae* S288C proteome was downloaded from the OrthoMCL database using the "scer" species identifier (53, 54). Mapping was performed by matching the ORF names in each data set. This was done through specific, *ad hoc*, pipelines written in the Perl programming language, yielding a set of primary parsed tab-delimited files that contained all pairs of gene-drug associations derived from the HIP profiles. All scripts and the standardized data set are publicly accessible through GitHub (https://github.com/trypanosomatics/yeast_to_tryp).

### Bioinformatic and chemoinformatic pipeline

A comparative chemogenomic pipeline was developed to map yeast genes to the corresponding *T. cruzi* orthologs and to further filter the data set of drug-target tuples. The pipeline runs as a consolidated Python program with modular functions designed to select data for each filter. It is publicly available as a Jupyter notebook in the GitHub repository. The chemical properties, such as hydrogen bond donors, hydrogen bond acceptors, molecular weight, and partition coefficient (log P), were calculated using RDKit (55). Marvin JS (MarvinSketch 21.7, 2021 ChemAxon) was used for drawing, displaying, and characterizing chemical structures (http://www.chemaxon.com). Automated classification of chemical compounds was done using Classyfire v2024 (56). The pipeline is publicly available through GitHub (see https://github.com/trypanosomatics/yeast_to_tryp).

### Chemical compounds and reagents

Benznidazole powder (Abarax, Laboratorio Elea Phoenix, Argentina) was dissolved in DMSO at 20 mM. Other experimental compounds were sourced from the following suppliers through Molport SIA (Riga, Latvia) and the Hit2Lead Chembridge Chemical stores (Table S2). All compounds were dissolved in DMSO at 20 mM, aliquoted, and stored at −20°C. All replicates were carried out using the same compound stock.

### Parasite culture

*T. cruzi* Tulahuen strain parasites constitutively expressing *Escherichia coli β*-galactosidase (Tul *β*-gal) were kindly supplied by Dr. Fred Buckner (University of Washington, USA). Infective trypomastigote stocks were expanded by serial passage in CF1 mice. At the time of maximal parasitemia, bloodstream trypomastigotes were harvested and purified by density gradient centrifugation using Ficoll in a swinging-bucket rotor, according to previously established procedures. Purified parasites were cryopreserved in liquid nitrogen for subsequent experimental use.

For infection assays, Tul *β*-gal expressing *T. cruzi* trypomastigotes were propagated in Vero cells cultured at 37°C under a humidified atmosphere containing 5% CO_2_. Host cells were maintained in minimum essential medium (MEM; Gibco) supplemented with 10% fetal bovine serum, penicillin (100 U/mL), and streptomycin (10 μg/mL). Cell-derived trypomastigotes were harvested from culture supernatants at 96 h post-infection and recovered by centrifugation at 5,000 × *g* for 10 min. Parasite cultures were routinely passaged in Vero cells for up to 40 passages without detectable reduction in *β*-galactosidase activity, after which fresh cultures were re-established from cryopreserved bloodstream trypomastigotes.

### Trypanocidal activity determination

To determine the trypanocidal activity of compounds, we set up a colorimetric assay that uses transgenic *T. cruzi* Tulahuen parasites expressing bacterial *β*-galactosidase (LacZ) (57). Enzyme activity (degradation of the chromogenic substrate chlorophenol red-*β*-D-galactopyranoside, CPRG) was used as a proxy for parasite growth, as described previously (57). Briefly, kidney epithelial cells (Vero cell lineage, from African green monkey) were seeded into 96-well plates at 5,000 cells/well. After 24 h, the cells were infected with 50,000 purified trypomastigotes. After 24 h of infection, the cultures were thoroughly washed with PBS to remove free trypomastigotes and incubated with RPMI medium without phenol red (Gibco Cat No. 11835030) with the addition of compounds at 20 μM. Benznidazole (BNZ) and voriconazole (VCZ), also at 20 μM, were used as positive controls, along with DMSO (0.5%) for carrier control, and infected/untreated and non-infected/untreated controls were used to obtain the assay’s maximum and minimum *β*-gal activity expected measurements. Screening assays were performed with two technical replicates and one biological replicate.

After culture incubation of cells in the presence of 20 μM of a given compound for 96 h, 100 μL of a freshly prepared PBS solution containing 1% NP40 and 100 μM CPRG (Roche, Cat No. 10884308001) was added to each well to reach a final concentration of 0.5% NP40 and 50 μM CPRG. Plates were then incubated for 4 h at 37°C and kept in the dark throughout the incubation. Finally, *β*-gal activity was determined by reading the absorbance at 595 nm using a plate reader (FilterMax F5 Multimode Microplate Reader, Molecular Devices).

Parasite growth inhibition was calculated using the following equation 1, where x denotes the absorbance measurement of the sample, IC is the absorbance of the infected control (maximum parasite growth), and NIC is the absorbance of the non-infected control (minimum parasite growth).


(1) $$\%\;Trypanocidal\;activity=\frac{x-NIC}{IC-NIC}*100$$

Compounds exhibiting ≥60% *T. cruzi* growth inhibition and ≥60% Vero cell viability at the screening concentration were classified as direct hits and subsequently evaluated in EC50 dose-response assays. Concentration-response assays to determine EC50 values were conducted in 96-well plates under the experimental conditions described above.

Test compounds were evaluated beginning at the maximum concentration used in the assay, followed by nine consecutive 2-fold serial dilutions. Each condition was assayed with two technical replicates and four biological replicates. The first biological replicate was performed using one batch of parasites. Biological replicates 2, 3, and 4 were performed using a new batch that was first passaged through mice and subsequently maintained in culture.

Trypanocidal activity was quantified according to equation 1, and the resulting data were analyzed using GraphPad Prism (version 8.0.1; GraphPad Software, LLC). EC50 values were derived for each compound by nonlinear regression of log-transformed concentration-response curves using a variable-slope model. The 95% confidence intervals (CI) for each EC50 were calculated by the same nonlinear regression analysis using data from all biological replicates.

### *β*-galactosidase inhibition assay

Active compounds were tested for inhibition of *β*-galactosidase activity to discard false-positive molecules interfering with the reporter assay. An extract of transgenic *T. cruzi* Tulahuen *β*-gal parasites was used as the source of *β*-galactosidase. Transgenic parasites expressing *β*-galactosidase were obtained from culture media of infected cells, as described. The supernatant from a cell culture flask was centrifuged at 5,700 × *g* for 10 min and washed twice with PBS. The pellet was resuspended in 100 μL of PBS, and then, 0.5% NP40 and 1 μM of E-64 proteinase inhibitor were added and incubated for 1 h at 37°C. After incubation, the parasite lysate was sonicated in four cycles of 5 s at an amplitude of 5%, followed by centrifugation at 16,200 × *g* for 30 min at 4°C. The supernatant was collected, supplemented with 20% glycerol, and aliquots were stored at −80°C until use.

Test compounds were incubated with *β*-galactosidase lysate (1:2,000 dilution) for 30 min at 37°C in activity buffer (PBS, 2% RPMI, 0.25% DMSO, 0.5% NP-40, pH 7.4) prior to the addition of 50 μM CPRG substrate. Data were collected and processed as described above. Residual enzyme activity was calculated using equation 1 to generate dose-response curves. IPTG (0–5 mM) was included as a *β*-galactosidase inhibition control.

### Cytotoxicity determination

To determine the cytotoxicity of compounds, we used a resazurin assay, which measures the conversion of resazurin to resorufin by living cells (58), using the same compound concentrations as described, on cultures of non-infected Vero cells. Cells were seeded at 5,000 cells/well, cultured for 48 h, washed with PBS, incubated with compound-supplemented fresh RPMI media, and incubated for 96 h. Next, 10 μL of a freshly prepared resazurin (RZ) 10× solution (440 μM in PBS) was added to each well. A DMSO 0.5% vehicle control was also included, along with a control of untreated cells and a blank control (no-cells) for maximum and minimum reagent reduction readings. The final reading was taken after 7 h of incubation in a black 96-well plate, and fluorescence (λ excitation/emission: 535 nm/595 nm) was recorded in a plate reader (FilterMax F5 Multimode Microplate Reader, Molecular Devices). All assays were performed with two technical replicates and one biological replicate. For the initial screening at fixed dose, the percent of cell viability was calculated using equation 2, where F is the fluorescence measurement and μC + represents the mean of vehicle controls.


(2) $$\%\;Cell\;viability=\frac{F}{\mu C+}*100$$

Compounds that preserved at least 60% viability in Vero cells at the screening concentration were classified as non-cytotoxic and prioritized for CC50 determination based on their *T. cruzi* growth inhibition profiles. Cytotoxicity dose-response assays were subsequently conducted under the same experimental conditions described above, using serial dilutions of each compound starting from 100 μM. All experimental conditions were performed with two technical replicates and four biological replicates. The resulting data were analyzed using GraphPad Prism (version 8.0.1; GraphPad Software LLC) by nonlinear regression of log-transformed concentration-response curves with a variable slope. CC50 values were calculated independently for each compound and defined as the concentration that reduced cell viability by 50%. For compounds that did not exhibit measurable cytotoxicity within the concentration range tested, CC50 values were reported as greater than the highest concentration assayed. Selectivity indices (SI) were calculated for each compound as the ratio between cytotoxicity (CC50) and antiparasitic activity (EC50).

### Compound comparison between CID 12 and known lysyl-tRNA synthetase inhibitors

Chemical structures were obtained from the related publication, and SMILES representations were obtained from Marvin JS (Chemaxon). Each molecule was processed using RDKit to generate both 2D and 3D molecular representations.

Pairwise atom-level comparisons were performed using RDKit’s Get Similarity Map For Fingerprint function to highlight the contribution of individual atoms and substructures to overall similarity (55). Two-dimensional fingerprint-based similarity metrics, including the Tanimoto and Braun-Blanquet coefficients, were calculated for each inhibitor relative to CID 12. Morgan fingerprints (radius 2, 2048 bits) were generated for each molecule using RDKit, and Tanimoto similarity was computed directly between the fingerprint of CID 12 and those of the known inhibitors (59). To complement this, atom-based fingerprints were generated using JCompoundMapper (ASP) (60), and Braun-Blanquet coefficients were calculated as the ratio of shared features to the maximum number of features in either molecule, implemented as a NumPy-based function operating on the explicit bit vectors derived from the fingerprints (61). Three-dimensional shape-based alignments were carried out using the O3A (Optimal 3D Alignment) method, with CID 12 as the reference molecule, and similarity was quantified using the Tversky index, which allows asymmetric weighting to emphasize either the reference or query molecule (62).

Similarity scores were interpreted using defined thresholds for 2D (Tanimoto and Braun-Blanquet) and 3D (Tversky) metrics, classifying pairs using predefined thresholds: values ≥ 0.75 were classified as high similarity, 0.60–0.74 as moderate-high similarity, 0.45–0.59 as moderate similarity, and <0.45 as low similarity. Visualizations of individual molecules, pairwise similarity maps, and combined 3D alignments were generated using RDKit to provide both quantitative and qualitative assessments of structural overlap, functional group conservation, and stereochemical correspondence among the compounds. The code to replicate this section can be found in a Python Jupyter notebook in the GitHub repository mentioned below.

## RESULTS

### Data resources for repurposing analysis

Five haploinsufficiency profiling (HIP) data sets from diverse *S. cerevisiae* chemogenomic assays were selected to build the predictive algorithm (Table 1). These data sets represent the current cumulative collection of chemogenomic screens performed on yeast. The data set from Giaever et al. (37) includes 80 genome-wide screens performed in pooled cultures of approximately 6,000 *S. cerevisiae* heterozygous deletion strains, using compounds with previously validated therapeutic targets across a range of concentrations (Table S1). The other data sets used the same pool of 6,000 HIP mutants to test novel compound libraries. The data set from Hillenmeyer et al. (38) had 726 diverse compounds, including the Food and Drug Administration-approved drugs and compounds with an unknown mechanism of action. The data set from Hoepfner et al. was obtained through the use of a fully automated robotic system, testing 1,800 compounds, 135 of which have well-established targets and 1,665 with unknown mechanisms of action, against a collection of 6,000 HIP mutants (40). The data set from Lee et al. was derived from a screening of 3,250 diverse drug-like small molecules that led to the identification of 317 compounds associated with a fitness defect in *S. cerevisiae*, 95% of which had an unknown target (41). Finally, the study by Wildenhain et al. had the largest number of compounds analyzed, with a total of 5,518 compounds, finding 1,964 active compounds (50).

### Predicting repurposing scenarios for trypanosomatids

**Fig 1:**
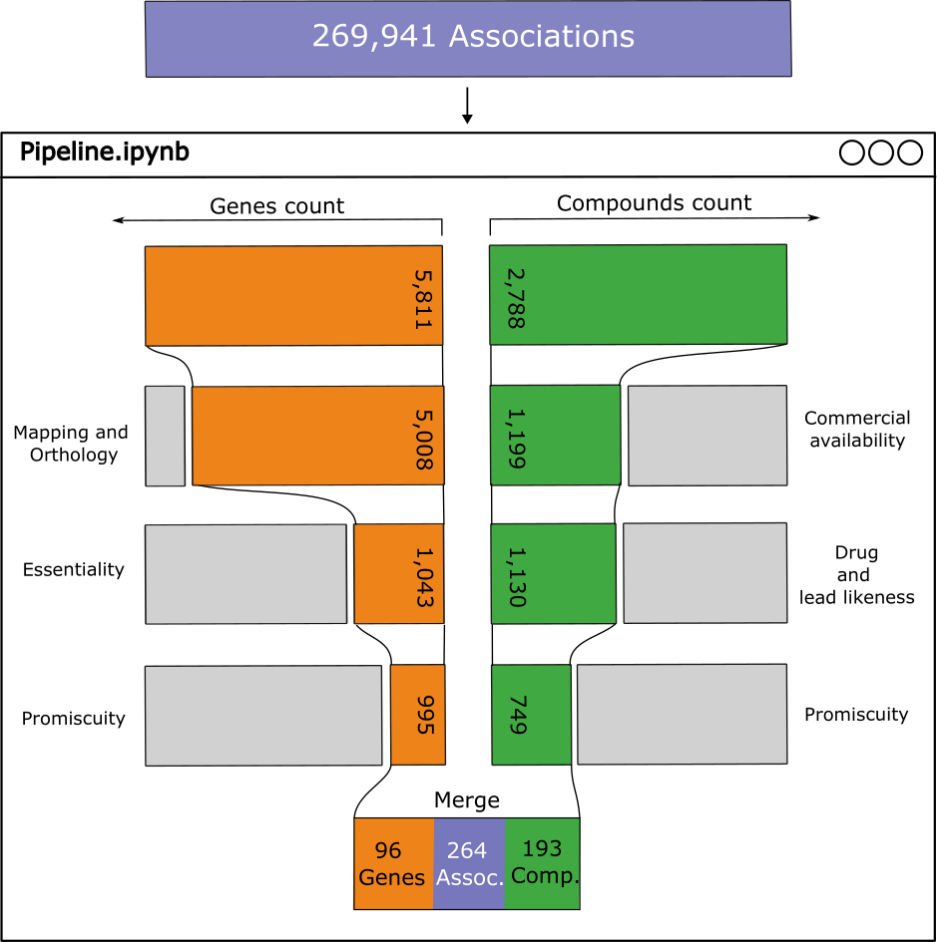
Workflow for compound and gene selection. An initial set of 2,788 compounds, 5,811 genes, and 269,941 tuples was sequentially filtered through several criteria. Compounds were first filtered by availability at Molport, followed by the evaluation of drug-likeness properties, and finally by promiscuity, defined as the number of genes associated with each compound. Genes were filtered based on orthology to *T. cruzi*, essentiality information in *T. brucei*, and promiscuity, defined as the number of compounds associated with each gene. In both cases, a promiscuity threshold was implemented to prioritize entities involved in lower cardinality relationships. After filtering, the final data set consisted of 193 compounds, 96 genes, and 264 unique compound-gene associations.

To prioritize repositioning candidates from the experimental drug-target tuples from yeast, a bioinformatic and chemoinformatic pipeline was designed (see Methods). The pipeline used the yeast drug-target tuples as input records and processed the data from drugs (chemoinformatic) and targets (proteins, bioinformatic) separately. The pipeline, schematically depicted in Fig. 1, processes all tuples in two parallel streams.

First, to identify conserved druggable modules between yeasts and trypanosomatids, we mapped yeast genes to the corresponding *T. cruzi* and *T. brucei* orthologs through assignment of OrthoMCL groups (53, 63). Only drug-target tuples whose genes shared an orthology group with these trypanosomatids were selected. Because this type of information is not available for *T. cruzi*, we used data on essential genes for *T. brucei* from a large-scale RNAi (knockdown) phenotyping screening (33) that is available in the TDR Targets database (64). The reduction in the number of drug-target tuples attained by these filters is shown in Fig. 1.

The drug information in the drug-target tuples was used in a separate chemoinformatic process. The first filter in this other path consisted of the selection of commercially available compounds in Molport, a chemical marketplace that sources compounds from many different primary providers (65). The second filter module consisted of selecting compounds with chemical properties akin to those of orally bioavailable drugs. Compounds were evaluated against Lipinski’s Rule of 5 and the Rule of 3, retaining those that met at least three of the four criteria in either set as drug-like; the rest were classified as non-drug-like (66, 67).

As described, all genes and drugs in our data were paired in the form of drug-target tuples, which represent an association derived from experimental chemogenomic screenings. Hence, for a given gene, there was a mapping to at least one chemical compound; likewise, any given compound was mapped to at least one gene. However, the cardinality of these associations was not limited to cases of one drug, one target.

Because the cardinality in these associations is related to the promiscuity of drugs (less specific mechanism of action), we implemented a promiscuity threshold in our pipeline for both genes and drugs. For genes, the threshold was set to retain only those associated with less than 200 drugs, and for drugs, it was set to keep the compounds associated with less than 20 genes.

**Fig 2:**
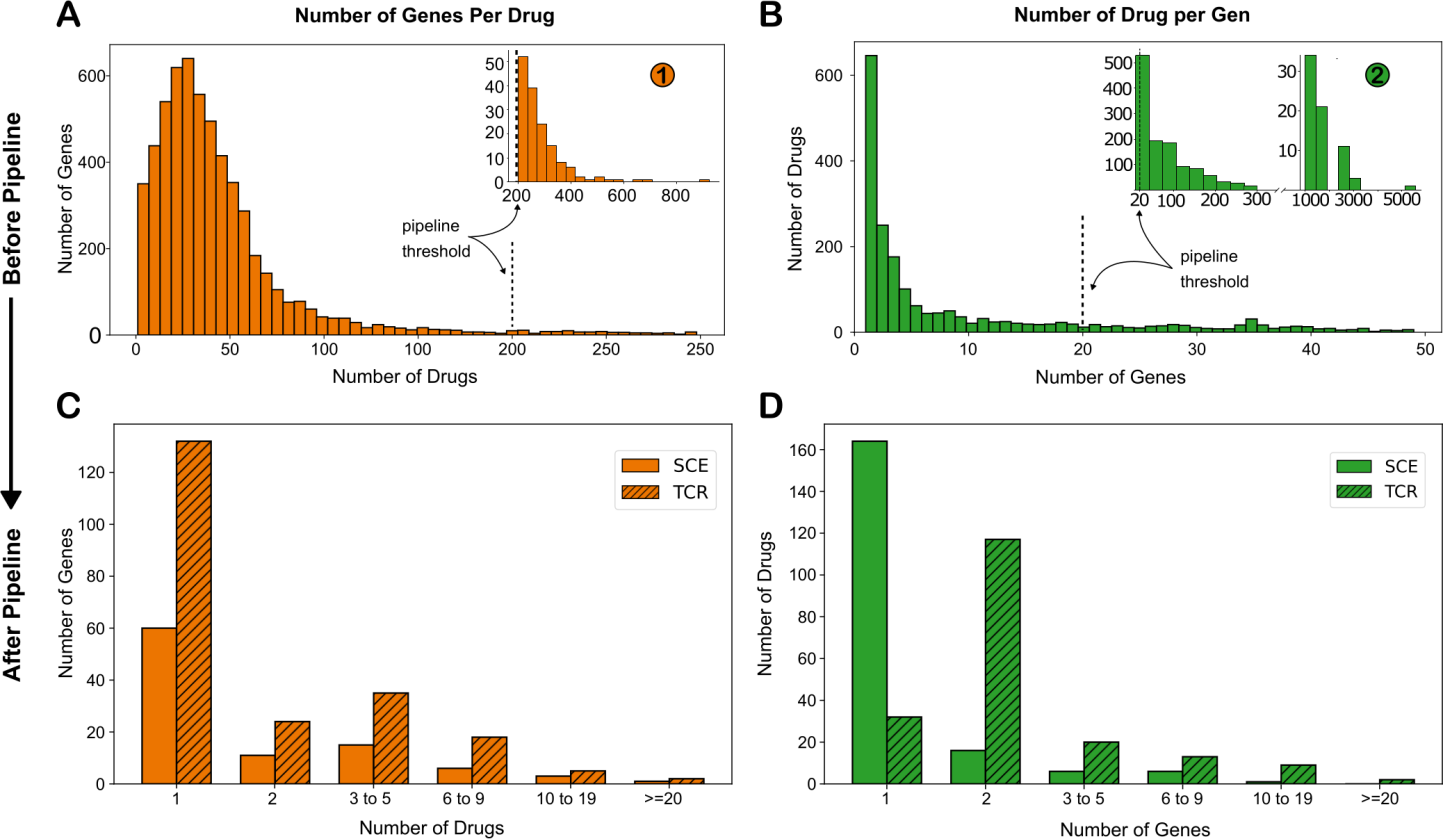
Cardinality distributions of compound-gene associations before and after the pipeline. Top row: distribution of yeast genes and their associated drugs before applying filters (**A and B**). Bottom row: distribution of yeast (SCE) and mapped *T. cruzi* (TCR) genes and their associated drugs after processing through the pipeline (**C and D**). Left column: histogram plots show the number of genes (y-axis) with drugs binned in the x-axis (**A and C**). Right column: histogram plots show the number of drugs (y-axis) with genes binned on the x-axis (**B and D**). The insets (1, 2) in the top plots show the same data but with different scales on the y-axis and different binning of drugs (orange) or genes (green) on the x-axis. In the top plots, dashed lines depict the threshold for filtering as implemented in the pipeline (A = 200 drugs; B = 20 genes).

Prior to filtering, some yeast genes (*n* = 154, Fig. 2A, see inset 1) were associated with more than 200 compounds, and conversely, some compounds (*n* = 1,155, Fig. 2B, inset 2) were linked to 20 or more genes. After applying the filtering pipeline, the distribution shifted: the majority of genes were now associated with only one (62.5%) or a few compounds (panels A vs. C). Similarly, the resulting distribution for drugs (Fig. 2D) was shifted to those with less than 10 associated target genes, with most compounds (83%) linked to a single gene (panels B vs. D). In the figure, panels C and D also display the equivalent distributions for the mapped *T. cruzi* orthologs. As expected, both distributions are quite similar to those from *S. cerevisiae* and are skewed to low cardinality associations, indicating that the pipeline effectively reduced potential non-specific or promiscuous elements. Unsurprisingly, the second most abundant category for genes in *T. cruzi* is “2” (panel D), while for *S. cerevisiae* (panel D), the most populated category is “1,” reflecting the existence of ∼ two gene variants for each yeast target in the hybrid genome of CL-Brener (68, 69).

After filtering, no compounds in *S. cerevisiae* were associated with more than 20 genes, confirming the effectiveness of the compound-level promiscuity filter in the yeast space. When mapping orthologs to the space of *T. cruzi* genes, only two compounds slightly exceeded this threshold (23 and 26 genes); however, these compounds were originally associated with only 9 and 10 yeast genes, respectively. At the gene level, a single yeast gene (YML094W, prefoldin subunit 5) was associated with 60 compounds, well below the gene-level cutoff (200), and this connectivity was propagated to two *T. cruzi* orthologs (TcCLB.506859.90 and TcCLB.511815.60).

As a result, we obtained a selection of 995 S. cerevisiae genes (815 ortholog groups, 1,737 *T. cruzi* [strain CL Brener] genes) with chemogenomic profiling data, which represent conserved orthologs between yeasts and trypanosomes. The final step consisted of merging the two parallel data streams to obtain the gene-drug pairs that had both genes and drugs selected by the bioinformatic and chemoinformatic streams. This list, containing 264 combinations, is available as supplementary data (Table S1).

### Curation and characterization of the resultant chemical library

The output of the modular pipeline was a list of 193 candidate compounds. To prioritize these, we performed a manual curation to further downscale the number of compounds to test experimentally. To inform the curation and selection process, we collected additional data from several sources, including (i) a commercial provider (Molport), (ii) the TDR Targets database, a representative bioinformatic resource integrating chemical and genomic information for human pathogens (64), (iii) PubChem, a large repository of chemical information (70), and (iv) SwissADME, an integrated set of tools and resources to calculate physicochemical descriptors and predict ADME parameters, pharmacokinetic properties, and medicinal chemistry friendliness of candidate compounds (71). Finally, we also performed bibliographic and web searches on all compounds using their IUPAC names and standard identifiers.

Some general criteria were used to deprioritize unsuitable candidates. For each compound, a search in TDR Targets was performed to navigate the chemical and genomic vicinity around these compounds and their targets. Using PubChem and TDR Targets as data sources, we collected information on reported assays for each compound. We reviewed PubChem bioassay data along with reported safety and hazard information. As part of our filtering criteria, compounds that had already been tested on trypanosomatids or that carried critical safety warnings were excluded. Finally, a bibliographic search was performed to exclude compounds whose substructures or functional groups had already been tested against trypanosomatids.

From the initial set of 193 compounds, 22 (11.4%) were selected as candidates for purchase following manual curation. A total of 100 compounds, representing 51.3% of the data set, were not selected due to an undefined or low-specificity rationale; however, these compounds may be investigated in future studies as new data or publications emerge, or through exploratory screening approaches. Among the compounds previously tested on trypanosomatids, 27 (14%) were discarded due to inactivity against *T. cruzi*, 13 (6.7%) because they did not show activity against other trypanosomatids; finally, 2 (1%) were excluded based on shared chemical scaffolds with compounds previously shown to be inactive against trypanosomatids. Regarding commercial availability, 23 compounds (11.9%) were not available for purchase, and 3 (1.6%) were excluded due to high cost (>$200 USD/mg). This outcome may reflect changes in compound availability between the initial pipeline filtering, which relied on supplier listings available at that time, and the subsequent manual curation. This represents a limitation of the pipeline, as fluctuations in commercial availability can bias candidate selection toward compounds that are currently purchasable and lead to the exclusion of potentially interesting candidates that are not listed by chemical suppliers at the time of analysis.

**Fig 3:**
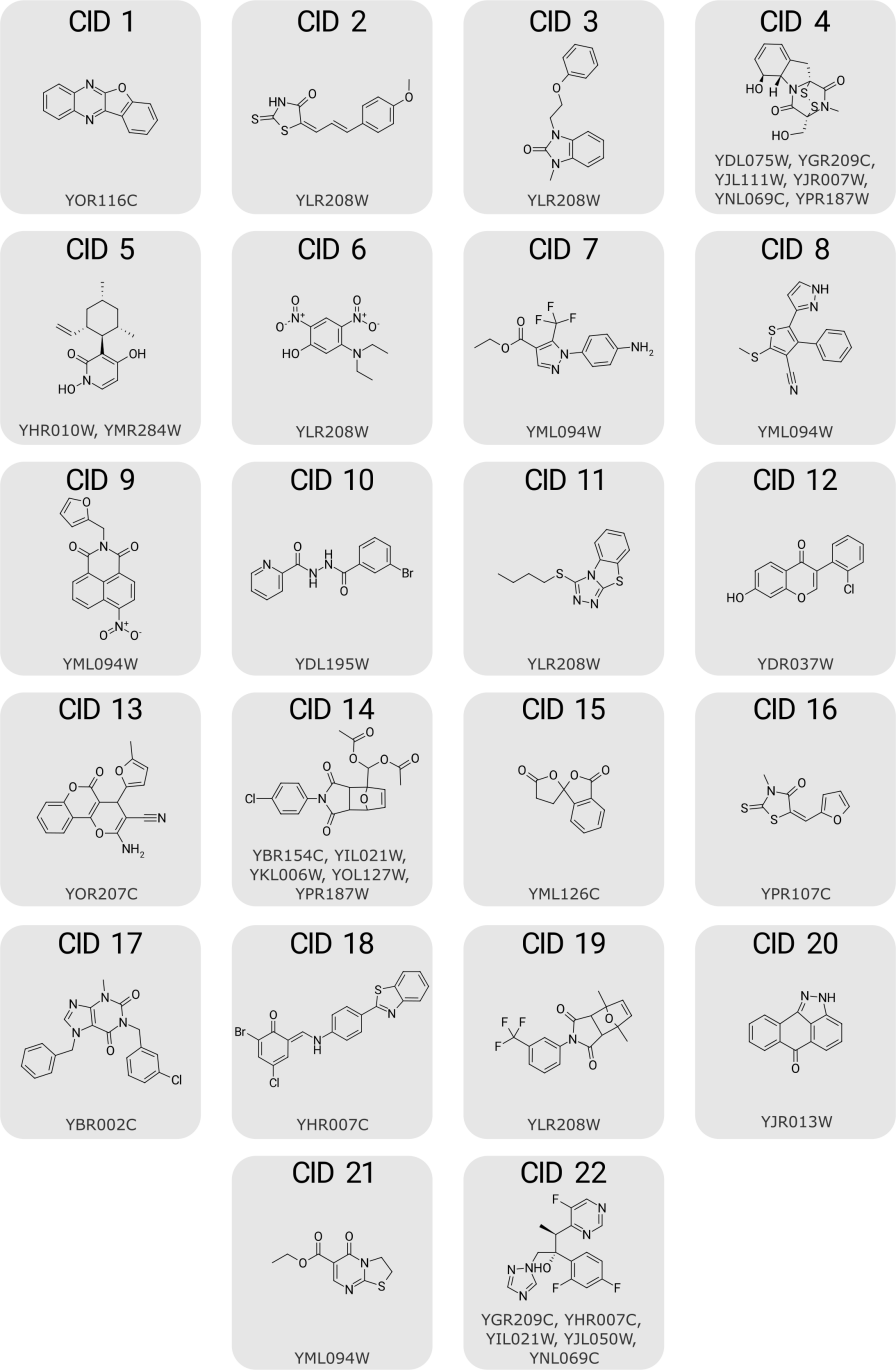
Depictions of compounds sourced and tested against *T. cruzi*. Each panel displays the chemical structure labeled with its CID (top) and the putative chemogenomic associations with *S. cerevisiae* gene target or targets (bottom).

We finally purchased 22 investigational compounds that meet the mentioned criteria (Fig. 3; Table S2). We additionally included voriconazole in this purchase as a positive control, as this is a compound that was part of the results from the pipeline but showed previous reports of activity on *T. cruzi* (72, 73). Voriconazole targets the fungal enzyme lanosterol 14α-demethylase (also known as CYP51), which is a key enzyme in the ergosterol biosynthesis pathway. This pathway is conserved between yeasts and trypanosomes, and it has been previously validated as a valid target for chemotherapeutic intervention (74), with several azoles tested and reaching clinical trials (30).

### Experimental validation of the pipeline for the discovery of anti-trypanosomal compounds

**Fig 4:**
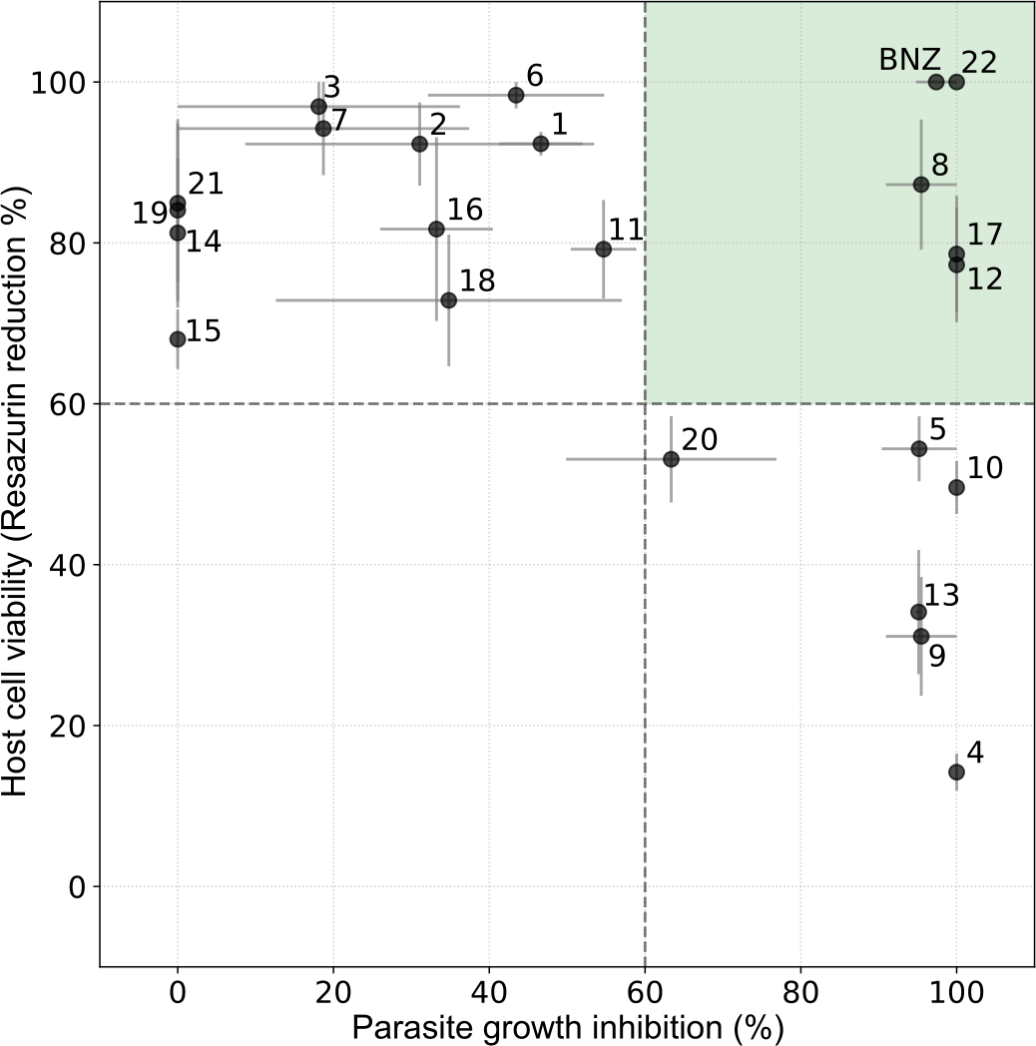
Screening of selected compounds from the repositioning pipeline. Scatter plot showing the percentage of parasite growth inhibition (x-axis) versus the percentage of host cell viability (y-axis) for compounds tested in the primary screening assay. Compounds were labeled with their internal IDs. The green-shaded area marks the region containing compounds with both high parasite inhibition (≥60%) and high host cell viability (≥60%), and prioritized for follow-up testing. Activity of benznidazole (BNZ) is shown as a reference. Each condition was measured in duplicate. For each data point, gray vertical and horizontal lines depict the variation across replicates.

In a primary screening, the 22 sourced compounds were tested *in vitro* to assess their cytotoxicity and trypanocidal activity at a fixed concentration of 20 μM (see Methods). The results are shown in Fig. 4. At the specified dose, 16 compounds showed low cytotoxicity on Vero cells (cellular viability ≥60%), and 10 compounds significantly inhibited *T. cruzi* replication (inhibition of parasite growth ≥60%) in a *T. cruzi* transgenic strain constitutively expressing the *E. coli β*-galactosidase as a reporter enzyme (57). These thresholds were set to select compounds with the highest potential to pass onto the secondary (dose-response) screening. Overall, four compounds showed the desired activity/selectivity profile (green box). Among them, the antifungal drug voriconazole (positive control, CID 22) was confirmed to be active in our assay, while the remaining were non-cytotoxic.

### Dose-response analysis of active compounds against *T. cruzi*

**Fig 5:**
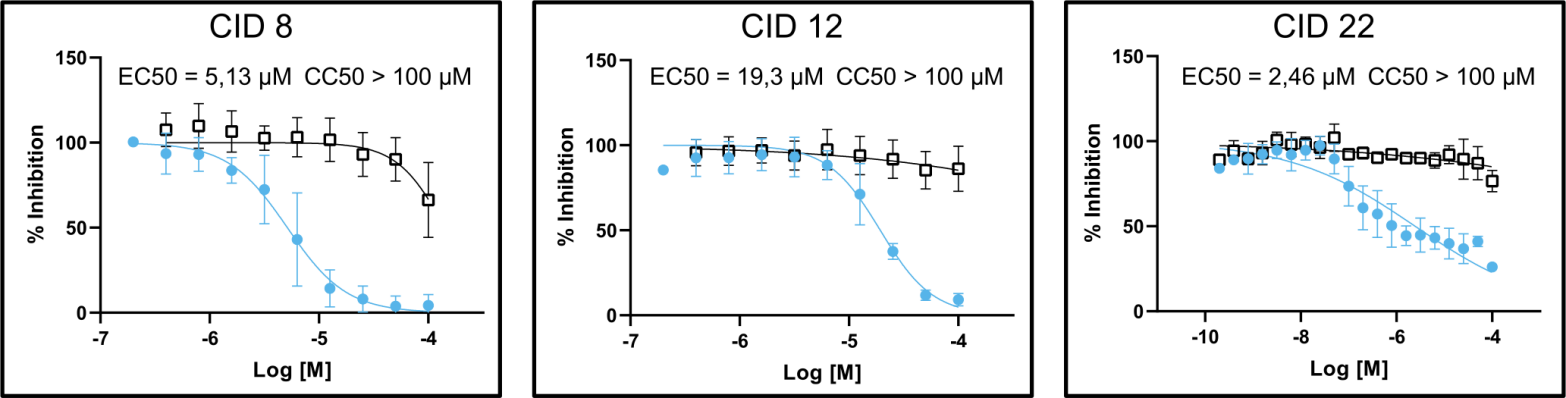
Dose-response curves for prioritized compounds. Selected compounds were assayed at different concentrations to obtain the depicted dose-response curves, showing parasite inhibition (light blue) and host cell viability (black) as a function of compound concentration. The corresponding EC50 (half-maximal effective concentration) and CC50 (half-maximal cytotoxic concentration) are shown inside the plots. EC50 and CC50 assays were performed using two technical replicates and four biological replicates.

The half-maximal effective concentration for the inhibition of parasite growth (EC50) and half-maximal cytotoxic concentration (CC50) for Vero cells were determined for the four selected compounds, and their selectivity indices (SI) were calculated as the EC50/CC50 ratio (Table 2). All the selected compounds displayed typical dose-response curves (Fig. 5), and EC50 values and the 95% confidence intervals (95% CI) are shown in Fig. S1.

**TABLE 2:** Summary of the anti-*T*. *cruzi* activity, cytotoxicity, and *β*-gal inhibitory activity of the selected compounds

CID	EC50 (M)	[95% CI]	CC50 (M)	Selectivity index (SI)	IC50 (M) *β*-Gal inhibition
CID 8	5.13 × 10^−6^	[4.47 × 10^−6^ − 5.88 × 10^−6^]	> 1.0 × 10^−4^	19.49	> 1.0 × 10^−4^
CID 12	1.93 × 10^−5^	[1.74 × 10^−5^ − 2.13 × 10^−5^]	> 1.0 × 10^−4^	5.18	> 1.0 × 10^−4^
CID 22	2.46 × 10^−6^	[1.64 × 10^−6^ − 3.85 × 10^−6^]	> 1.0 × 10^−4^	40.65	> 1.0 × 10^−4^

As expected, voriconazole (CID 22) confirmed its previously reported anti-*T. cruzi* activity (72, 73), allowing us to validate the feasibility of our computational pipeline. An EC50 value of 7.15 nM has been reported in the literature for the Tulahuen strain using an indirect assay based on trypomastigote release. In contrast, our results yielded a low-micromolar EC50 value (2.46 μM). The difference in magnitude between these values is likely attributable to the distinct experimental approaches employed.

Among the novel compounds identified, CID 8 exhibited the most potent antiparasitic activity, with an EC50 value of 5.13 μM. In the range of concentration tested, this compound showed only a modest cytotoxic effect at the highest concentration that did not reach 50% inhibition, resulting in a selectivity index value of 19.49. Compound CID 12 also displayed a favorable SI, although with comparatively lower antiparasitic potency. Finally, CID 17 exhibited trypanocidal activity in the initial screening and in the first biological replicate of the EC50 assay; however, this activity was not reproduced in subsequent independent biological replicates, indicating a possible loss of compound activity during (>2 years) storage.

All hit compounds were analyzed for pan-assay interference patterns, and none contained PAINS structural alerts (75). Finally, the selected compounds were tested in an enzymatic triage to discard those able to interfere with the reporter enzymatic methodology (*β*-galactosidase inhibitors) used in the screening assay. At the maximal concentration tested, none of these compounds showed detectable *β*-galactosidase inhibition (Fig. S2). Overall, these results validate the novel hits CID 8 and CID 12, selected from the repositioning pipeline, as *bona fide* hits with the ability to inhibit *T. cruzi* replication in phenotypic screenings.

## DISCUSSION

Chagas disease remains one of the major health problems in Latin America and worldwide, and the discovery of new antiprotozoal agents, with novel and safer modes of action, is urgently needed. In this work, we reused data from yeast haploinsufficiency mutants to provide chemogenomic associations to putative targets in the genome of *T. cruzi*. These represent good starting points to identify new candidate hits as they provide both a testable drug and a putative target.

### Research outputs

As a result of this work, we provide a number of outputs that can be used and improved upon by others. First, we have extracted and organized the extensive data on chemogenomic screens on HIP and HOP yeast mutants and provided both data sets, as well as the scripts used to parse and standardize it. Second, we provide one example of possible filtering criteria that can be used to map candidate yeast targets to other species using orthology and to downsample the resulting set of drug-target tuples. Third, we provide a chemical library that represents an enriched source of bioactive compounds with the potential to inhibit *T. cruzi* replication. A significant proportion of investigational compounds fail to translate activity from *in silico* or *in vitro* studies to *in cellulo* assays because they cannot effectively reach their (i.e., intracellular) targets (76, 77). By having both activity against *S. cerevisiae* and links to defined and meaningful protein targets based on associations derived from the screen of these drugs against haploinsufficient mutants, this strategy simultaneously reduces the chance of failure and provides verifiable parsimonious hypotheses about their putative targets/modes of action in the parasite.

The bioinformatic and chemoinformatic pipelines implemented in this work allowed us to reduce the original chemogenomic data set from 269,941 to 264 drug-target associations (0.09%). The criteria we used to filter and downsample drug-target associations were based on previously explored ideas and concepts (78, 79). While not providing a significant reduction, the mapping of orthologs was required to ensure proper assessment of candidate targets in *T. cruzi* and the possible mechanism of action of bioactive compounds. The most restrictive filtering criterion was the use of *T. brucei* essentiality data, based on high-throughput RNA interference phenotyping (33). Although *T. brucei* and *T. cruzi* have distinct lifestyles within their hosts, with the former being predominantly extracellular in the bloodstream and interstitial tissues (80) and the latter an intracellular parasite (81), this filtering step was essential to ensure that the selection of candidate genes reflected biologically meaningful targets and processes relevant to parasite survival. As discussed below, the targets of the identified hit compounds are conserved druggable modules affecting essential processes not associated with any particular lifestyle.

For compounds, the commercial availability in Molport was the most restrictive criterion. Although the compounds assayed in yeast chemogenomic screens were all available through different providers and hence qualify as being “commercially available,” we implemented further filtering criteria through Molport for practical reasons. First, Molport acts as a consolidation hub, sourcing compounds from many upstream chemical suppliers. As a result, the chemical library produced by our pipeline can be easily sourced from a single vendor. Second, Molport provides its database of compounds to perform chemical similarity searches either through their API or by direct download (65), with additional essential information for each compound (e.g., price range, in stock, and lead time if not in stock). Hence, this introduced a quick, programmatic way to guide further selection of compounds based on cost. However, while this provided a practical way to filter compounds, it is also a criterion with some variability over time, mostly based on changes in stocks, which introduces a non-deterministic process in the pipeline.

Other filtering criteria (lead-like chemical features and promiscuity) provided only marginal reductions in the size of the resulting chemical library, highlighting the good profile of the original libraries used in the yeast chemogenomic screenings. The code and parameters are available for others to adjust to their own settings or analyze the yeast chemogenomic data against other organisms.

### Active hits against *T. cruzi* and their putative targets: CID 12-lysyl-tRNA synthetase

The compound 12 is a 3-(2-chlorophenyl)-7-hydroxy-4H-chromen-4-one and was assayed in a study by Lee et al. (41). The proposed target, as reported by the *S. cerevisiae* chemogenomic screens, was the lysyl-tRNA synthetase encoded by the KRS1 gene (YDR037W). Our pipeline mapped this gene to the *T. cruzi* putative lysyl-tRNA synthetase encoded in the CL-Brener genome by the TcCLB.508971.30 gene (OrthoMCL OG5_126972 ortholog group).

Aminoacyl-tRNA synthetases are recognized as good candidate drug targets against diverse pathogens, including eukaryotic parasites (82–84) and mycobacteria (85). Specifically, lysyl-tRNA synthetase was identified in *Plasmodium falciparum* as the target of the fungal secondary metabolite cladosporin, with an estimated IC50 value in the nanomolar range (45). Similarly, compounds DDD508 and DDD489 act on lysyl-tRNA synthetase in *Cryptosporidium parvum* (84). In *Mycobacterium tuberculosis*, compound DDD02049209 has been identified as an effective inhibitor of the same enzyme (85). Finally, inhibitors of lysyl-tRNA synthetase have been shown to have efficacy against *T. cruzi* and the related trypanosomatid parasites *T. brucei* and *Leishmania donovani*, including *in vivo* studies, proposing lysyl-tRNA synthetase as a validated drug target in *T. cruzi* (86) (See Supplementary section 1).

Our pipeline (built using OrthoMCL v5) identified TcCLB.508971.30 as a potential target because it shares the orthology group OG5_126972 with the reported *S. cerevisiae* gene YDR037W. In *T. cruzi*, however, there are two additional lysyl-tRNA synthetase genes, TcCLB.503815.20 and TcCLB.505807.120. These genes belong to a different orthology group, OG5_147566, that does not include any *S. cerevisiae* members. In *T. brucei*, two distinct lysyl-tRNA synthetase coding sequences exist: TbKRS1 (Tb927.8.1600), which localizes to the cytoplasm and shares high identity with apicomplexan orthologs, and TbKRS2 (Tb927.6.1510), a mitochondrial variant that aligns more closely with bacterial enzymes (87). Phylogenetic reconstruction placed TcCLB.508971.30 and YDR037W within a clade that includes the cytoplasmic counterparts from *T. brucei*, while the genes TcCLB.503815.20 and TcCLB.505807.120 were clustered with the mitochondrial *T. brucei* counterpart (Fig. S3). This result supports their evolutionary relatedness and subcellular specialization and suggests that the orthology-based mapping correctly captured this and that TcCLB.508971.30 is the most closely related paralog to the yeast gene found in the chemogenomic profiling data.

Ultimately, the anti-trypanosomal activity and favorable selectivity index of CID 12 validate our comparative chemogenomic approach, demonstrating its effectiveness in successfully repositioning drug-target associations from yeast models to identify novel hits against *T. cruzi*. This success is substantiated by the precise mapping of the cytoplasmic ortholog TcCLB.508971.30 (OG5_126972), which correctly mirrors the chemogenomic profile of the yeast gene YDR037W, and by the structural consistency of its chromen-4-one scaffold, which shares a rigid hetero-bicyclic core and significant 3D similarity with other potent lysyl-tRNA synthetase inhibitors.

### Active hits against *T. cruzi* and their putative targets: CID 8-prefoldin

Regarding compound 8, the yeast chemogenomic assay of Wildenhain et al. showed the gene GimC/YML094W, prefoldin (subunit 5) as the target (50). Our pipeline mapped this gene to the *T. cruzi* putative prefoldin orthologs encoded in the CL-Brener genome as TcCLB.506859.90 and TcCLB.511815.60.

This protein has a hetero-hexameric structure with a wide range of functions, the main one being a chaperone (88, 89). It has been shown that the protein or the subunits are involved in proteasomal degradation of misfolded proteins, initiation of translation machinery assembly, RNA polymerase biogenesis, DNA repair, regulation of gene expression, and transcript elongation, among others (90). The alterations in this protein cause the imbalance of many cellular processes, like tumor growth or neurodegenerative diseases in humans (91).

Most compounds reported against this protein were identified using the screening assay described by Wildenhain et al., with 13 classified as active and 87 as inactive. From the active compounds, a multi-target disruption of several yeast cellular processes was observed (Table S3). Specifically, inhibition of dolichyl-P-mannosyltransferases (YNR030W) disrupts protein glycosylation, targeting serine/threonine kinases (YNL298W, YHR030C, and YLR113W), interferes with cell cycle regulation and stress signaling, and perturbation of cytoskeletal and motor proteins (YEL061C and YML094W) affecting intracellular transport and morphogenesis. In addition, inhibition of phosphatases (YDL134C) and chromatin-associated factors (YLR085C) modulates signaling and gene expression, collectively impairing yeast growth and viability.

It has been reported that the loss of this gene causes a disbalance in many biological processes (90). Hence, when assessing the fitness profile of GimC haploinsufficient mutants, it cannot be ruled out that CID 8 is targeting another effector in one of the multiple affected pathways. In fact, most other prefoldin subunits in the starting chemogenomic data were discarded in the promiscuity of the pipeline due to the large number of reported associations (Fig. S4).

### Caveats and considerations regarding candidate targets

It should also be acknowledged that the selection process was influenced by an additional, non-scientific consideration: the commercial availability of compounds. Although this practical filter is essential for facilitating downstream validation, it necessarily means that some compounds (including those that may be structurally related or even more potent) were omitted simply because they could not be obtained commercially.

Another limitation of our study is that we focused on the most obvious/suitable candidates: that is, only those compounds displaying low cytotoxicity and high anti-*T*. *cruzi* activity at 20 μM were selected to progress to the dose-response secondary screening. There are, in addition, six compounds under the cytotoxicity threshold, five of which inhibited almost completely *T. cruzi* replication in culture at this concentration. Despite the fact that their apparent potency might be influenced by cytotoxicity itself (i.e., given that *T. cruzi* is an intracellular parasite, the reduction in the number of viable cells might artificially increase the observed antiparasitic effect), there is still a chance to find useful compounds from this subset, potentially increasing the success rate of our computational approach.

Finally, the associations reported in chemogenomic assays are inferred from the loss of fitness of pools of mutants and do not provide direct information about the binding/interaction of the tested compounds with their associated target(s). Although these experiments provide important physiological clues about the mode of action of a compound, and preliminary evidence about their putative targets in yeast (37), their effective engagement/implication needs to be further validated in independent experiments. In line with this, the same approach needs to be followed with the derived ortholog genes in *T. cruzi*, the most parsimonious candidates for the observed antiparasitic action of the compounds.

The prioritized targets include the cytoplasmic lysyl-tRNA synthetase (TcKRS, TcCLB.508971.30), which requires precise differentiation from its mitochondrial paralogs and subunit 5 of prefoldin (TcCLB.506859.90 and TcCLB.511815.60), a hetero-hexameric chaperone whose complex multi-subunit structure and diverse roles in protein homeostasis make validating direct inhibition particularly difficult. To confirm the essentiality and relevance of these candidates, several molecular biology approaches in *T. cruzi* are proposed, including CRISPR/Cas9-mediated gene knockout (92), inducible gene knockdown (93), and protein overexpression (94) to monitor changes in drug sensitivity, as well as direct enzymatic assays using recombinant TcKRS (or yeast KRS) to measure CID 12 inhibition *in vitro*.

### Conclusions

Through the use of a bioinformatic and chemoinformatic modular pipeline, we repositioned compounds with associated yeast chemogenomic profiling data to trypanosomatids. This was mostly based on the identification of conserved orthologs between yeasts and trypanosomes and the transference of associated drugs from one to another. The modular pipeline developed in this work was tailored to downsample these libraries based on additional reasonable criteria. The selection of these filtering criteria can be adapted to the needs of other projects, providing versatility to manage the number, chemical, and biological properties of the resultant compounds. In this work, we chose to favor a pragmatic approach to quickly arrive at a small set enriched in novel, drug-like, and non-promiscuous compounds that, acting through essential/biologically relevant targets for trypanosomatids, could be commercially sourced at a reasonable cost.

Importantly, the prioritized list of 193 compounds (Table S3) may be a useful resource to initiate a systematic phenotypic analysis to identify anti-*T*. *cruzi* agents with potentially novel mechanisms of action. In this sense, expanding the experimental evaluation may render new active compounds—and their candidate molecular targets—to be added to the ones validated here. The initial activity observed for CID 17 could not be consistently replicated across subsequent biological replicates; therefore, retesting this compound using freshly prepared stocks may help determine whether its trypanocidal activity can be reproduced. Also, exploring further the chemical space around CID 8 and CID 12 active scaffolds may provide more potent candidates that exhibit even lower cytotoxicity. Finally, the success rate attained by reusing chemogenomic profiling data from another microorganism is similar to or better than the rates achieved by other approaches (95, 96). This indicates that the developed pipeline can help fuel the identification of suitable drug candidates for Chagas disease or other parasitic infections.

Beyond the immediate findings, this study delivers reusable resources and starting points for future drug discovery efforts. The underlying yeast chemogenomic data set was carefully curated and standardized, enabling its direct reuse and reanalysis in the context of other pathogens or biological systems without additional preprocessing. Moreover, although only 22 compounds from the prioritized 193-compound library were experimentally evaluated here, the remaining candidates are commercially available and can be readily tested to further expand the phenotypic and mechanistic landscape of anti-*T*. *cruzi* activity. Finally, the two validated active compounds identified in this work provide concrete chemical starting points for subsequent medicinal chemistry optimization, target validation, and lead development, supporting the broader applicability of this pipeline as an efficient entry into antiparasitic drug discovery.

## Data Availability

All molecular structures and their activities are available in machine-readable format in the Supplemental material. All yeast chemogenomic data, curated and integrated from their original publications, as well as all methods, and software described are publicly accessible under a BSD-style open-source license in an associated repository (https://github.com/trypanosomatics/yeast_to_tryp).

## SUPPLEMENTAL MATERIAL

Supplemental figures aac.00117-26-s0001.pdf Fig. S1 to S5.

Supplemental tables aac.00117-26-s0002.xlsx Tables S1 to S4.

ASM does not own the copyrights to Supplemental Material that may be linked to, or accessed through, an article. The authors have granted ASM a non-exclusive, world-wide license to publish the Supplemental Material files. Please contact the corresponding author directly for reuse.

## References

[1] De Rycker M, Wyllie S, Horn D, Read KD, Gilbert IH. 2023. Anti-trypanosomatid drug discovery: progress and challenges. Nat Rev Microbiol 21:35–50. doi:10.1038/s41579-022-00777-y 35995950 PMC9395782

[2] Rassi A Jr, Rassi A, Marin-Neto JA. 2010. Chagas disease. The Lancet 375:1388–1402. doi:10.1016/S0140-6736(10)60061-X 20399979

[3] Pérez-Molina JA, Molina I. 2018. Chagas disease. The Lancet 391:82–94. doi:10.1016/S0140-6736(17)31612-4 28673423

[4] Büscher P, Cecchi G, Jamonneau V, Priotto G. 2017. Human African trypanosomiasis. The Lancet 390:2397–2409. doi:10.1016/S0140-6736(17)31510-6 28673422

[5] Burza S, Croft SL, Boelaert M. 2018. Leishmaniasis. The Lancet 392:951–970. doi:10.1016/S0140-6736(18)31204-2 30126638

[6] Buscaglia CA, Kissinger JC, Agüero F. 2015. Neglected tropical diseases in the post-genomic era. Trends Genet 31:539–555. doi:10.1016/j.tig.2015.06.002 26450337

[7] Beyrer C, Villar JC, Suwanvanichkij V, Singh S, Baral SD, Mills EJ. 2007. Neglected diseases, civil conflicts, and the right to health. The Lancet 370:619–627. doi:10.1016/S0140-6736(07)61301-4 17707757

[8] Brindley PJ, Hotez PJ, Kamhawi S. 2025. Revisiting what constitutes a neglected tropical disease? PLoS Negl Trop Dis 19:e0012794. doi:10.1371/journal.pntd.0012794 39977425 PMC11841861

[9] World Health Organization. 2024. World health statistics 2024: monitoring health for the SDGs, sustainable development goals. World Health Organization (WHO), Geneva, Switzerland.

[10] Hotez PJ, Molyneux DH, Fenwick A, Kumaresan J, Sachs SE, Sachs JD, Savioli L. 2007. Control of neglected tropical diseases. N Engl J Med 357:1018–1027. doi:10.1056/NEJMra064142 17804846

[11] Trouiller P, Olliaro P, Torreele E, Orbinski J, Laing R, Ford N. 2002. Drug development for neglected diseases: a deficient market and a public-health policy failure. The Lancet 359:2188–2194. doi:10.1016/S0140-6736(02)09096-7 12090998

[12] Pérez-Molina JA, Crespillo-Andújar C, Bosch-Nicolau P, Molina I. 2021. Trypanocidal treatment of Chagas disease. Enferm Infecc Microbiol Clin (Engl Ed) 39:458–470. doi:10.1016/j.eimce.2020.04.012 34736749

[13] Hughes J, Rees S, Kalindjian S, Philpott K. 2011. Principles of early drug discovery. Br J Pharmacol 162:1239–1249. doi:10.1111/j.1476-5381.2010.01127.x 21091654 PMC3058157

[14] Mulcahy A, Rennane S, Schwam D, Dickerson R, Baker L, Shetty K. 2025. Use of clinical trial characteristics to estimate costs of new drug development. JAMA Netw Open 8:e2453275. doi:10.1001/jamanetworkopen.2024.53275 39761045 PMC11704977

[15] Adams CP, Brantner VV. 2006. Estimating the cost of new drug development: is it really $802 million? Health Aff (Millwood) 25:420–428. doi:10.1377/hlthaff.25.2.420 16522582

[16] Wyatt PG, Gilbert IH, Read KD, Fairlamb AH. 2011. Target validation: linking target and chemical properties to desired product profile. Curr Top Med Chem 11:1275–1283. doi:10.2174/156802611795429185 21401506 PMC3182078

[17] Hernandez JJ, Pryszlak M, Smith L, Yanchus C, Kurji N, Shahani VM, Molinski SV. 2017. Giving drugs a second chance: overcoming regulatory and financial hurdles in repurposing approved drugs as cancer therapeutics. Front Oncol 7:273. doi:10.3389/fonc.2017.00273 29184849 PMC5694537

[18] Jones LH, Bunnage ME. 2017. Applications of chemogenomic library screening in drug discovery. Nat Rev Drug Discov 16:285–296. doi:10.1038/nrd.2016.244 28104905

[19] Müller S, Ackloo S, Al Chawaf A, Al-Lazikani B, Antolin A, Baell JB, Beck H, Beedie S, Betz UAK, Bezerra GA, et al.. 2022. Target 2035 – update on the quest for a probe for every protein. RSC Med Chem 13:13–21. doi:10.1039/D1MD00228G 35211674 PMC8792830

[20] Hernandez HW, Soeung M, Zorn KM, Ashoura N, Mottin M, Andrade CH, Caffrey CR, de Siqueira-Neto JL, Ekins S. 2019. High throughput and computational repurposing for neglected diseases. Pharm Res 36:27. doi:10.1007/s11095-018-2558-3 PMC6792295 30560386

[21] Andrade CH, Neves BJ, Melo-Filho CC, Rodrigues J, Silva DC, Braga RC, Cravo PVL. 2019. In silico chemogenomics drug repositioning strategies for neglected tropical diseases. Curr Med Chem 26:4355–4379. doi:10.2174/0929867325666180309114824 29521204

[22] Ojo KK, Arakaki TL, Napuli AJ, Inampudi KK, Keyloun KR, Zhang L, Hol WGJ, Verlinde CLMJ, Merritt EA, Van Voorhis WC. 2011. Structure determination of glycogen synthase kinase-3 from Leishmania major and comparative inhibitor structure-activity relationships with Trypanosoma brucei GSK-3. Mol Biochem Parasitol 176:98–108. doi:10.1016/j.molbiopara.2010.12.009 21195115 PMC3045540

[23] Ruberto I, Szoor B, Clark R, Matthews KR. 2013. Investigating mammalian tyrosine phosphatase inhibitors as potential “Piggyback” leads to target Trypanosoma brucei transmission. Chem Biol Drug Des 81:291–301. doi:10.1111/cbdd.12079 23066974 PMC4003393

[24] Porta EOJ, Kalesh K, Steel PG. 2023. Navigating drug repurposing for chagas disease: advances, challenges, and opportunities. Front Pharmacol 14:1233253. doi:10.3389/fphar.2023.1233253 37576826 PMC10416112

[25] Cosentino RO, Agüero F. 2014. Genetic profiling of the isoprenoid and sterol biosynthesis pathway genes of Trypanosoma cruzi. PLoS One 9:e96762. doi:10.1371/journal.pone.0096762 24828104 PMC4020770

[26] Didier Garnham M, Agüero FA, Ramírez JC, Agüero F, Salas-Sarduy E. 2026. Identification of antifungal agents AR-12 and fosmanogepix as anti-Trypanosoma cruzi drugs through an enhanced fluorogenic β-galactosidase phenotypic screening assay. ACS Infect Dis 12:724–737. doi:10.1021/acsinfecdis.5c00900 41479158

[27] Kumari S, Kumar V, Tiwari RK, Ravidas V, Pandey K, Kumar A. 2022. Amphotericin B: a drug of choice for Visceral Leishmaniasis. Acta Trop 235:106661. doi:10.1016/j.actatropica.2022.106661 35998680

[28] de Macedo-Silva ST, de Souza W, Rodrigues JCF. 2015. Sterol biosynthesis pathway as an alternative for the anti-protozoan parasite chemotherapy. Curr Med Chem 22:2186–2198. doi:10.2174/0929867322666150319120337 25787966

[29] Molina I, Gómez i Prat J, Salvador F, Treviño B, Sulleiro E, Serre N, Pou D, Roure S, Cabezos J, Valerio L, Blanco-Grau A, Sánchez-Montalvá A, Vidal X, Pahissa A. 2014. Randomized trial of posaconazole and benznidazole for chronic Chagas’ disease. N Engl J Med 370:1899–1908. doi:10.1056/NEJMoa1313122 24827034

[30] Torrico F, Gascon J, Ortiz L, Alonso-Vega C, Pinazo M-J, Schijman A, Almeida IC, Alves F, Strub-Wourgaft N, Ribeiro I, E1224 Study Group. 2018. Treatment of adult chronic indeterminate Chagas disease with benznidazole and three E1224 dosing regimens: a proof-of-concept, randomised, placebo-controlled trial. Lancet Infect Dis 18:419–430. doi:10.1016/S1473-3099(17)30538-8 29352704 PMC7612561

[31] Machado PRL, Rosa MEA, Guimarães LH, Prates FVO, Queiroz A, Schriefer A, Carvalho EM. 2015. Treatment of disseminated leishmaniasis with liposomal amphotericin B. Clin Infect Dis 61:945–949. doi:10.1093/cid/civ416 26048961

[32] Alrajhi AA, Ibrahim EA, De Vol EB, Khairat M, Faris RM, Maguire JH. 2002. Fluconazole for the treatment of cutaneous leishmaniasis caused by Leishmania major. N Engl J Med 346:891–895. doi:10.1056/NEJMoa011882 11907288

[33] Alsford S, Turner DJ, Obado SO, Sanchez-Flores A, Glover L, Berriman M, Hertz-Fowler C, Horn D. 2011. High-throughput phenotyping using parallel sequencing of RNA interference targets in the African trypanosome. Genome Res 21:915–924. doi:10.1101/gr.115089.110 21363968 PMC3106324

[34] Collett CF, Kitson C, Baker N, Steele-Stallard HB, Santrot M-V, Hutchinson S, Horn D, Alsford S. 2019. Chemogenomic profiling of antileishmanial efficacy and resistance in the related kinetoplastid parasite Trypanosoma brucei. Antimicrob Agents Chemother 63:e00795-19. doi:10.1128/AAC.00795-19 31160283 PMC6658743

[35] Pradhan A, Siwo GH, Singh N, Martens B, Balu B, Button-Simons KA, Tan A, Zhang M, Udenze KO, Jiang RHY, Ferdig MT, Adams JH, Kyle DE. 2015. Chemogenomic profiling of Plasmodium falciparum as a tool to aid antimalarial drug discovery. Sci Rep 5:15930. doi:10.1038/srep15930 26541648 PMC4635350

[36] Giaever G, Shoemaker DD, Jones TW, Liang H, Winzeler EA, Astromoff A, Davis RW. 1999. Genomic profiling of drug sensitivities via induced haploinsufficiency. Nat Genet 21:278–283. doi:10.1038/6791 10080179

[37] Giaever G, Flaherty P, Kumm J, Proctor M, Nislow C, Jaramillo DF, Chu AM, Jordan MI, Arkin AP, Davis RW. 2004. Chemogenomic profiling: identifying the functional interactions of small molecules in yeast. Proc Natl Acad Sci USA 101:793–798. doi:10.1073/pnas.0307490100 14718668 PMC321760

[38] Hillenmeyer ME, Fung E, Wildenhain J, Pierce SE, Hoon S, Lee W, Proctor M, St Onge RP, Tyers M, Koller D, Altman RB, Davis RW, Nislow C, Giaever G. 2008. The chemical genomic portrait of yeast: uncovering a phenotype for all genes. Science 320:362–365. doi:10.1126/science.1150021 18420932 PMC2794835

[39] Hillenmeyer ME, Ericson E, Davis RW, Nislow C, Koller D, Giaever G. 2010. Systematic analysis of genome-wide fitness data in yeast reveals novel gene function and drug action. Genome Biol 11:R30. doi:10.1186/gb-2010-11-3-r30 20226027 PMC2864570

[40] Hoepfner D, Helliwell SB, Sadlish H, Schuierer S, Filipuzzi I, Brachat S, Bhullar B, Plikat U, Abraham Y, Altorfer M, et al.. 2014. High-resolution chemical dissection of a model eukaryote reveals targets, pathways and gene functions. Microbiol Res 169:107–120. doi:10.1016/j.micres.2013.11.004 24360837

[41] Lee AY, St Onge RP, Proctor MJ, Wallace IM, Nile AH, Spagnuolo PA, Jitkova Y, Gronda M, Wu Y, Kim MK, et al.. 2014. Mapping the cellular response to small molecules using chemogenomic fitness signatures. Science 344:208–211. doi:10.1126/science.1250217 24723613 PMC4254748

[42] Chen Y, Mallick J, Maqnas A, Sun Y, Choudhury BI, Côte P, Yan L, Ni T, Li Y, Zhang D, Rodríguez-Ortiz R, Lv Q, Jiang Y, Whiteway M. 2018. Chemogenomic profiling of the fungal pathogen Candida albicans. Antimicrob Agents Chemother 62. doi:10.1128/AAC.02365-17 PMC5786791 29203491

[43] Parsons AB, Brost RL, Ding H, Li Z, Zhang C, Sheikh B, Brown GW, Kane PM, Hughes TR, Boone C. 2004. Integration of chemical-genetic and genetic interaction data links bioactive compounds to cellular target pathways. Nat Biotechnol 22:62–69. doi:10.1038/nbt919 14661025

[44] Ericson E, Gebbia M, Heisler LE, Wildenhain J, Tyers M, Giaever G, Nislow C. 2008. Off-target effects of psychoactive drugs revealed by genome-wide assays in yeast. PLoS Genet 4:e1000151. doi:10.1371/journal.pgen.1000151 18688276 PMC2483942

[45] Hoepfner D, McNamara CW, Lim CS, Studer C, Riedl R, Aust T, McCormack SL, Plouffe DM, Meister S, Schuierer S, Plikat U, Hartmann N, Staedtler F, Cotesta S, Schmitt EK, Petersen F, Supek F, Glynne RJ, Tallarico JA, Porter JA, Fishman MC, Bodenreider C, Diagana TT, Movva NR, Winzeler EA. 2012. Selective and specific inhibition of the Plasmodium falciparum lysyl-tRNA synthetase by the fungal secondary metabolite cladosporin. Cell Host Microbe 11:654–663. doi:10.1016/j.chom.2012.04.015 22704625 PMC3391680

[46] Kapitzky L, Beltrao P, Berens TJ, Gassner N, Zhou C, Wüster A, Wu J, Babu MM, Elledge SJ, Toczyski D, Lokey RS, Krogan NJ. 2010. Cross-species chemogenomic profiling reveals evolutionarily conserved drug mode of action. Mol Syst Biol 6:451. doi:10.1038/msb.2010.107 21179023 PMC3018166

[47] Silberberg Y, Kupiec M, Sharan R. 2016. Utilizing yeast chemogenomic profiles for the prediction of pharmacogenomic associations in humans. Sci Rep 6:23703. doi:10.1038/srep23703 27025271 PMC4812343

[48] Barazandeh M, Kriti D, Nislow C, Giaever G. 2022. The cellular response to drug perturbation is limited: comparison of large-scale chemogenomic fitness signatures. BMC Genomics 23:197. doi:10.1186/s12864-022-08395-x 35277135 PMC8915488

[49] Hoepfner D, Helliwell SB, Sadlish H, Schuierer S, Filipuzzi I, Brachat S, Bhullar B, Plikat U, Abraham Y, Altorfer M, et al.. 2014. Data from: High-resolution chemical dissection of a model eukaryote reveals targets, pathways and gene functions. Dryad. doi:10.5061/dryad.v5m8v 24360837

[50] Wildenhain J, Spitzer M, Dolma S, Jarvik N, White R, Roy M, Griffiths E, Bellows DS, Wright GD, Tyers M. 2016. Systematic chemical-genetic and chemical-chemical interaction datasets for prediction of compound synergism. Sci Data 3:160095. doi:10.1038/sdata.2016.95 27874849 PMC5127411

[51] National Cancer Institute. 2016. NCI/CADD group chemoinformatics tools and user services. Available from: https://cactus.nci.nih.gov/. Retrieved 25 May 2026.

[52] Kim S, Thiessen PA, Cheng T, Yu B, Bolton EE. 2018. An update on PUG-REST: RESTful interface for programmatic access to PubChem. Nucleic Acids Res 46:W563–W570. doi:10.1093/nar/gky294 29718389 PMC6030920

[53] Li L, Stoeckert CJ Jr, Roos DS. 2003. OrthoMCL: identification of ortholog groups for eukaryotic genomes. Genome Res 13:2178–2189. doi:10.1101/gr.1224503 12952885 PMC403725

[54] Alvarez-Jarreta J, Amos B, Aurrecoechea C, Bah S, Barba M, Barreto A, Basenko EY, Belnap R, Blevins A, Böhme U, et al.. 2024. VEuPathDB: the eukaryotic pathogen, vector and host bioinformatics resource center in 2023. Nucleic Acids Res 52:D808–D816. doi:10.1093/nar/gkad1003 37953350 PMC10767879

[55] Landrum G, Tosco P, Kelley B, Rodriguez R, Cosgrove D, Vianello R, Gedeck P, Jones G, Kawashima E, Schneider N, et al.. 2025. Rdkit/rdkit: 2025_09_4 (Q3 2025) release. Zenodo. doi:10.5281/ZENODO.18098214

[56] Djoumbou Feunang Y, Eisner R, Knox C, Chepelev L, Hastings J, Owen G, Fahy E, Steinbeck C, Subramanian S, Bolton E, Greiner R, Wishart DS. 2016. ClassyFire: automated chemical classification with a comprehensive, computable taxonomy. J Cheminform 8:61. doi:10.1186/s13321-016-0174-y 27867422 PMC5096306

[57] Buckner FS, Verlinde CL, La Flamme AC, Van Voorhis WC. 1996. Efficient technique for screening drugs for activity against Trypanosoma cruzi using parasites expressing beta-galactosidase. Antimicrob Agents Chemother 40:2592–2597. doi:10.1128/AAC.40.11.2592 8913471 PMC163582

[58] Ansar Ahmed S, Gogal RM Jr, Walsh JE. 1994. A new rapid and simple non-radioactive assay to monitor and determine the proliferation of lymphocytes: an alternative to [3H]thymidine incorporation assay. J Immunol Methods 170:211–224. doi:10.1016/0022-1759(94)90396-4 8157999

[59] Bajusz D, Rácz A, Héberger K. 2015. Why is Tanimoto index an appropriate choice for fingerprint-based similarity calculations? J Cheminform 7:20. doi:10.1186/s13321-015-0069-3 26052348 PMC4456712

[60] Hinselmann G, Rosenbaum L, Jahn A, Fechner N, Zell A. 2011. jCompoundMapper: an open source Java library and command-line tool for chemical fingerprints. J Cheminform 3:3. doi:10.1186/1758-2946-3-3 21219648 PMC3033338

[61] Safizadeh H, Simpkins SW, Nelson J, Li SC, Piotrowski JS, Yoshimura M, Yashiroda Y, Hirano H, Osada H, Yoshida M, Boone C, Myers CL. 2021. Improving measures of chemical structural similarity using machine learning on chemical-genetic interactions. J Chem Inf Model 61:4156–4172. doi:10.1021/acs.jcim.0c00993 34318674 PMC8479812

[62] Horvath D, Marcou G, Varnek A. 2013. Do not hesitate to use Tversky-and other hints for successful active analogue searches with feature count descriptors. J Chem Inf Model 53:1543–1562. doi:10.1021/ci400106g 23731338

[63] Chen F, Mackey AJ, Vermunt JK, Roos DS. 2007. Assessing performance of orthology detection strategies applied to eukaryotic genomes. PLoS One 2:e383. doi:10.1371/journal.pone.0000383 17440619 PMC1849888

[64] Urán Landaburu L, Berenstein AJ, Videla S, Maru P, Shanmugam D, Chernomoretz A, Agüero F. 2019. TDR targets 6: driving drug discovery for human pathogens through intensive chemogenomic data integration. Nucleic Acids Res:gkz999. doi:10.1093/nar/gkz999 PMC7145610 31680154

[65] Molport. 2026. Research chemical suppliers, catalogs and a searchable molecule database. Molport. Available from: https://www.molport.com. Retrieved 25 May 2026.

[66] Lipinski CA, Lombardo F, Dominy BW, Feeney PJ. 2001. Experimental and computational approaches to estimate solubility and permeability in drug discovery and development settings. Adv Drug Deliv Rev 46:3–26. doi:10.1016/s0169-409x(00)00129-0 11259830

[67] Congreve M, Carr R, Murray C, Jhoti H. 2003. A “rule of three” for fragment-based lead discovery? Drug Discov Today 8:876–877. doi:10.1016/s1359-6446(03)02831-9 14554012

[68] Andersson B. 2011. The Trypanosoma cruzi genome; conserved core genes and extremely variable surface molecule families. Res Microbiol 162:619–625. doi:10.1016/j.resmic.2011.05.003 21624458

[69] Chiurillo MA, Lander N. 2021. The long and winding road of reverse genetics in Trypanosoma cruzi. Microb Cell 8:203–207. doi:10.15698/mic2021.09.758 34527719 PMC8404153

[70] Kim S, Chen J, Cheng T, Gindulyte A, He J, He S, Li Q, Shoemaker BA, Thiessen PA, Yu B, Zaslavsky L, Zhang J, Bolton EE. 2021. PubChem in 2021: new data content and improved web interfaces. Nucleic Acids Res 49:D1388–D1395. doi:10.1093/nar/gkaa971 33151290 PMC7778930

[71] Daina A, Michielin O, Zoete V. 2017. SwissADME: a free web tool to evaluate pharmacokinetics, drug-likeness and medicinal chemistry friendliness of small molecules. Sci Rep 7:42717. doi:10.1038/srep42717 28256516 PMC5335600

[72] Gulin J E N, Eagleson MA, Postan M, Cutrullis RA, Freilij H, Bournissen FG, Petray PB, Altcheh J. 2013. Efficacy of voriconazole in a murine model of acute Trypanosoma cruzi infection. J Antimicrob Chemother 68:888–894. doi:10.1093/jac/dks478 23212113

[73] Gulin JEN, Eagleson MA, López-Muñoz RA, Solana ME, Altcheh J, García-Bournissen F. 2020. In vitro and in vivo activity of voriconazole and benznidazole combination on Trypanosoma cruzi infection models. Acta Trop 211:105606. doi:10.1016/j.actatropica.2020.105606 32598923

[74] Urbina JA, Docampo R. 2003. Specific chemotherapy of Chagas disease: controversies and advances. Trends Parasitol 19:495–501. doi:10.1016/j.pt.2003.09.001 14580960

[75] Baell JB, Holloway GA. 2010. New substructure filters for removal of pan assay interference compounds (PAINS) from screening libraries and for their exclusion in bioassays. J Med Chem 53:2719–2740. doi:10.1021/jm901137j 20131845

[76] Gülden M, Mörchel S, Seibert H. 2001. Factors influencing nominal effective concentrations of chemical compounds in vitro: cell concentration. Toxicol In Vitro 15:233–243. doi:10.1016/s0887-2333(01)00008-x 11377096

[77] Sun D, Gao W, Hu H, Zhou S. 2022. Why 90% of clinical drug development fails and how to improve it? Acta Pharm Sin B 12:3049–3062. doi:10.1016/j.apsb.2022.02.002 35865092 PMC9293739

[78] Crowther GJ, Shanmugam D, Carmona SJ, Doyle MA, Hertz-Fowler C, Berriman M, Nwaka S, Ralph SA, Roos DS, Van Voorhis WC, Agüero F. 2010. Identification of attractive drug targets in neglected-disease pathogens using an in silico approach. PLoS Negl Trop Dis 4:e804. doi:10.1371/journal.pntd.0000804 20808766 PMC2927427

[79] Magarinos MP, Carmona SJ, Crowther GJ, Ralph SA, Roos DS, Shanmugam D, Van Voorhis WC, Aguero F. 2012. TDR Targets: a chemogenomics resource for neglected diseases. Nucleic Acids Res 40:D1118–D1127. doi:10.1093/nar/gkr1053 22116064 PMC3245062

[80] Crilly NP, Mugnier MR. 2021. Thinking outside the blood: perspectives on tissue-resident Trypanosoma brucei. PLoS Pathog 17:e1009866. doi:10.1371/journal.ppat.1009866 34529724 PMC8445408

[81] Barrett MP, Burchmore RJ, Stich A, Lazzari JO, Frasch AC, Cazzulo JJ, Krishna S. 2003. The trypanosomiases. The Lancet 362:1469–1480. doi:10.1016/S0140-6736(03)14694-6 14602444

[82] Pham JS, Dawson KL, Jackson KE, Lim EE, Pasaje CFA, Turner KEC, Ralph SA. 2014. Aminoacyl-tRNA synthetases as drug targets in eukaryotic parasites. Int J Parasitol Drugs Drug Resist 4:1–13. doi:10.1016/j.ijpddr.2013.10.001 24596663 PMC3940080

[83] Nasim F, Qureshi IA. 2023. Aminoacyl tRNA synthetases: implications of structural biology in drug development against trypanosomatid parasites. ACS Omega 8:14884–14899. doi:10.1021/acsomega.3c00826 37151504 PMC10157851

[84] Caldwell N, Peet C, Miller P, Colon BL, Taylor MG, Cocco M, Dawson A, Lukac I, Teixeira JE, Robinson L, et al.. 2024. Cryptosporidium lysyl-tRNA synthetase inhibitors define the interplay between solubility and permeability required to achieve efficacy. Sci Transl Med 16:eadm8631. doi:10.1126/scitranslmed.adm8631 39441903 PMC7617456

[85] Green SR, Davis SH, Damerow S, Engelhart CA, Mathieson M, Baragaña B, Robinson DA, Tamjar J, Dawson A, Tamaki FK, et al.. 2022. Lysyl-tRNA synthetase, a target for urgently needed M. tuberculosis drugs. Nat Commun 13:5992. doi:10.1038/s41467-022-33736-5 36220877 PMC9552147

[86] Tulloch LB, Tawell H, Taylor AE, Lima ML, Dawson A, Carvalho S, Wall RJ, Corpas-Lopez V, Dey G, Duggan J, et al.. 2025. Antitrypanosomal quinazolines targeting lysyl-tRNA synthetase show partial efficacy in a mouse model of acute Chagas disease. Sci Transl Med 17:eadu4564. doi:10.1126/scitranslmed.adu4564 40632837 PMC7617978

[87] Español Y, Thut D, Schneider A, de Pouplana LR. 2009. A mechanism for functional segregation of mitochondrial and cytosolic genetic codes. Proc Natl Acad Sci USA 106:19420–19425. doi:10.1073/pnas.0909937106 19880741 PMC2780774

[88] Vainberg IE, Lewis SA, Rommelaere H, Ampe C, Vandekerckhove J, Klein HL, Cowan NJ. 1998. Prefoldin, a chaperone that delivers unfolded proteins to cytosolic chaperonin. Cell 93:863–873. doi:10.1016/s0092-8674(00)81446-4 9630229

[89] Liang J, Xia L, Oyang L, Lin J, Tan S, Yi P, Han Y, Luo X, Wang H, Tang L, Pan Q, Tian Y, Rao S, Su M, Shi Y, Cao D, Zhou Y, Liao Q. 2020. The functions and mechanisms of prefoldin complex and prefoldin-subunits. Cell Biosci 10:87. doi:10.1186/s13578-020-00446-8 32699605 PMC7370476

[90] Tahmaz I, Shahmoradi Ghahe S, Topf U. 2021. Prefoldin function in cellular protein homeostasis and human diseases. Front Cell Dev Biol 9:816214. doi:10.3389/fcell.2021.816214 35111762 PMC8801880

[91] Herranz-Montoya I, Park S, Djouder N. 2021. A comprehensive analysis of prefoldins and their implication in cancer. iScience 24:103273. doi:10.1016/j.isci.2021.103273 34761191 PMC8567396

[92] Peng D, Kurup SP, Yao PY, Minning TA, Tarleton RL. 2014. CRISPR-Cas9-mediated single-gene and gene family disruption in Trypanosoma cruzi. mBio 6:e02097-14. doi:10.1128/mBio.02097-14 25550322 PMC4281920

[93] Wen LM, Xu P, Benegal G, Carvaho MR, Butler DR, Buck GA. 2001. Trypanosoma cruzi: exogenously regulated gene expression. Exp Parasitol 97:196–204. doi:10.1006/expr.2001.4612 11384163

[94] Kelly JM, Ward HM, Miles MA, Kendall G. 1992. A shuttle vector which facilitates the expression of transfected genes in Trypanosoma cruzi and Leishmania. Nucleic Acids Res 20:3963–3969. doi:10.1093/nar/20.15.3963 1324472 PMC334073

[95] Peña I, Pilar Manzano M, Cantizani J, Kessler A, Alonso-Padilla J, Bardera AI, Alvarez E, Colmenarejo G, Cotillo I, Roquero I, de Dios-Anton F, Barroso V, Rodriguez A, Gray DW, Navarro M, Kumar V, Sherstnev A, Drewry DH, Brown JR, Fiandor JM, Julio Martin J. 2015. New compound sets identified from high throughput phenotypic screening against three kinetoplastid parasites: an open resource. Sci Rep 5:8771. doi:10.1038/srep08771 25740547 PMC4350103

[96] Buckner FS, Buchynskyy A, Nagendar P, Patrick DA, Gillespie JR, Herbst Z, Tidwell RR, Gelb MH. 2020. Phenotypic drug discovery for human African trypanosomiasis: a powerful approach. Trop Med Infect Dis 5:23. doi:10.3390/tropicalmed5010023 32033395 PMC7157241

