# Supplementary material for: New hit compounds against Trypanosoma cruzi derived from mining yeast chemogenomic profiling data
Source: Antimicrob Agents Chemother. 2026 Jun 4;70(7):e00117-26. doi: 10.1128/aac.00117-26 (PMC13321824; doi:10.1128/aac.00117-26)
Supplement: Supplemental figures — Fig. S1 to S5. [file aac.00117-26-s0001.pdf]

SUPPLEMENTARY MATERIALS for

New hit compounds against *Trypanosoma cruzi* derived from mining yeast chemogenomic profiling data

Mercedes Didier Garnham, Lionel Urán Landaburu, Emir Salas Sarduy and Fernán Agüero

Corresponding authors: esalas@iib.unsam.edu.ar, fernan@iib.unsam.edu.ar

This PDF file includes:

Figures S1 to S5

Supplementary section 1

Code, Software, Data:

Additional software (scripts, python notebook) and data (yeast chemogenomic reference datasets) are available from the associated Github repository: https://github.com/trypanosomatics/yeast_to_tryp

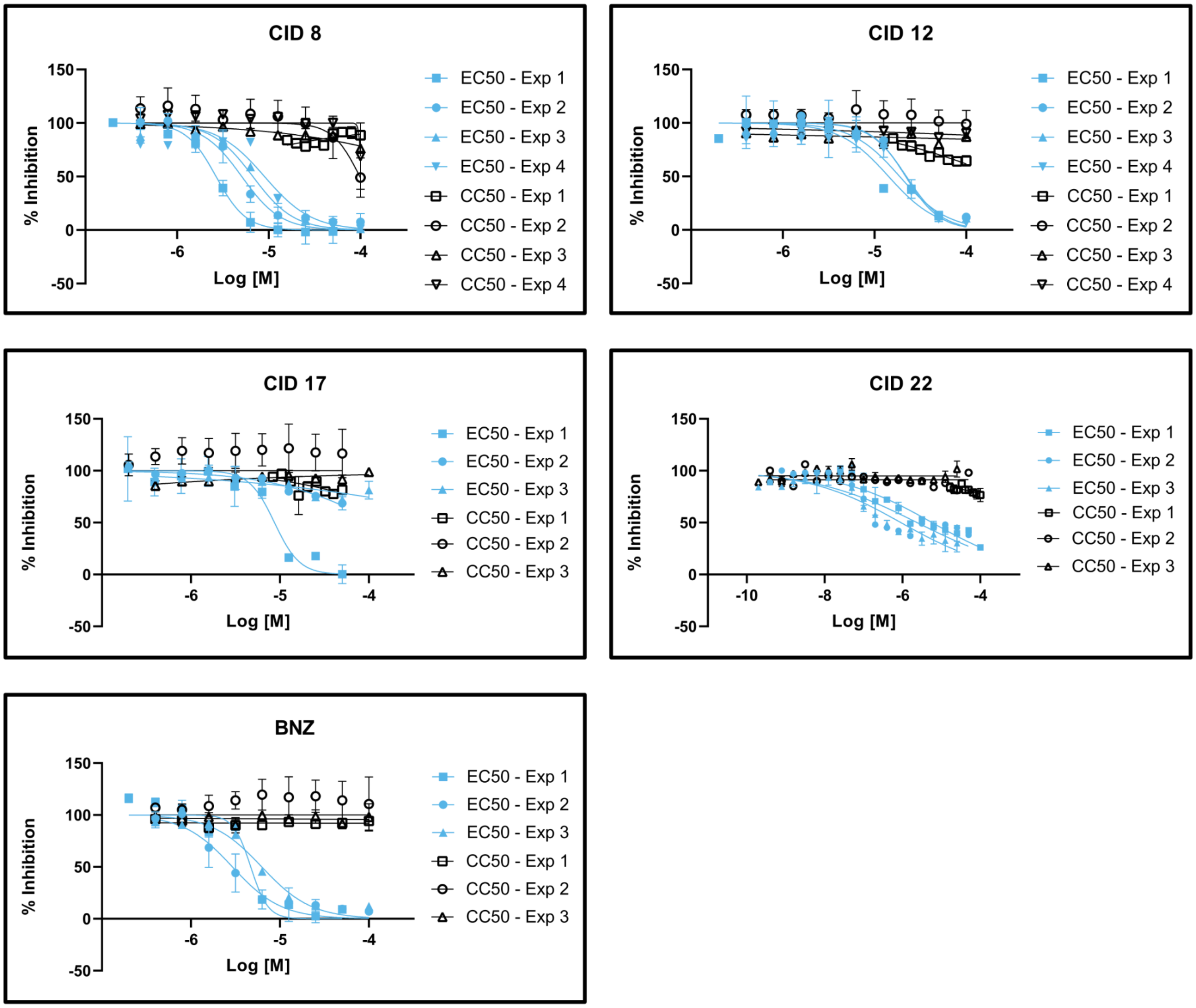

Figure S1. Dose–response curves from independent biological replicates.
Dose–response analyses for CID 8, CID 12, CID 17, CID 22, and BNZ. Each curve represents an independent biological replicate, with symbols corresponding to individual experiments. Data points are shown as mean values of technical replicates. Light blue curves correspond to EC50 determinations for trypanocidal activity, whereas black curves correspond to CC50 determinations for cytotoxicity in Vero cells. Curves were fitted using nonlinear regression.

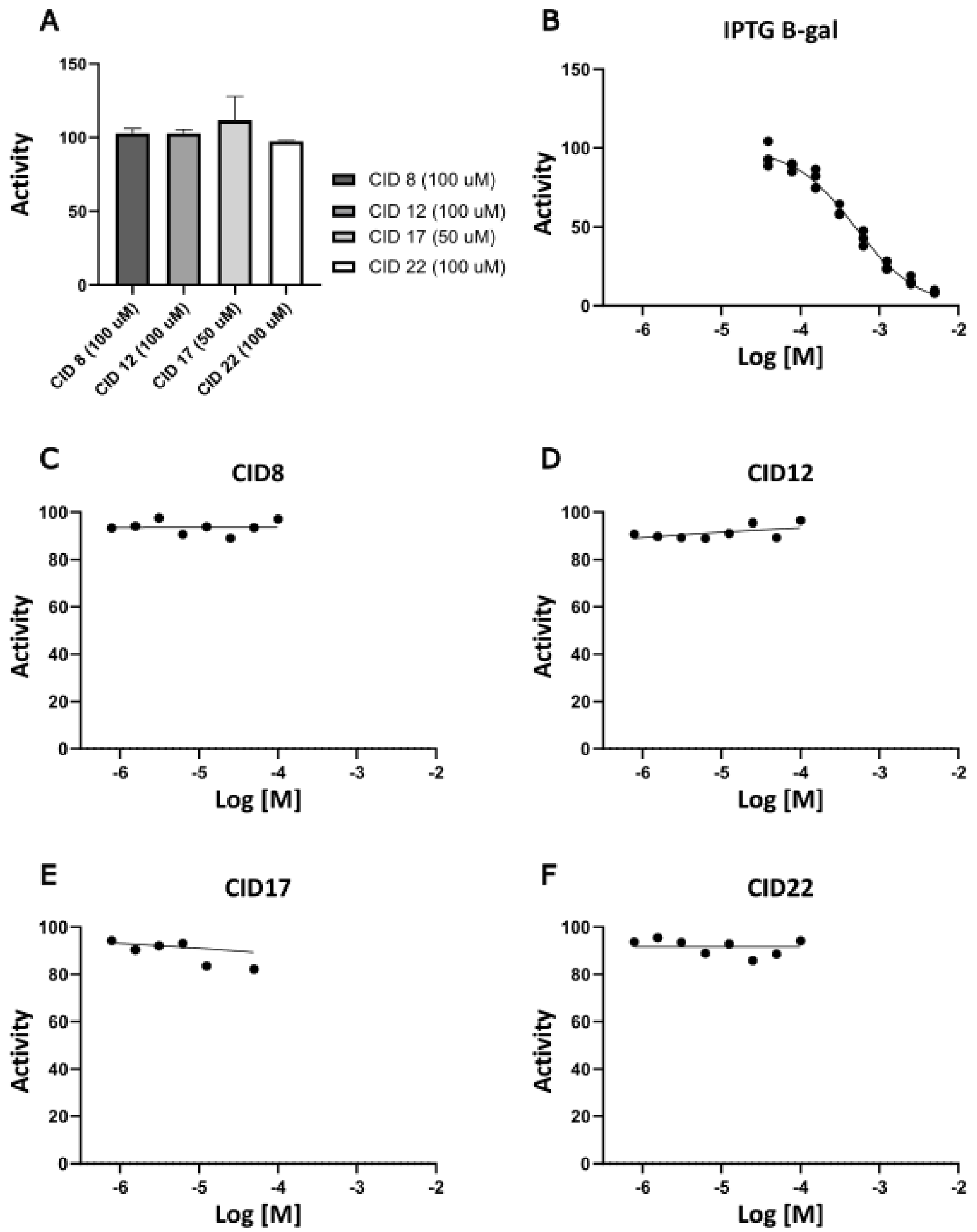

Figure S2. Results of the β-galactosidase inhibition assays. Active compounds were evaluated for β-galactosidase inhibition using parasite lysates. IPTG was included as a positive control. (A) β-Galactosidase activity was measured at a fixed concentration of CID17 (50 μM), CID8 (100 μM), CID12 (100 μM), and CID22 (100 μM). (B) Dose–response curve of IPTG, confirming assay sensitivity. Panels A and B correspond to one experiment performed with three technical replicates. (C–F) Dose–response analyses of CID8 (C), CID12 (D), CID17 (E), and CID22 (F). Each of these panels corresponds to one experiment performed with a single replicate. None of the tested compounds showed inhibitory activity against β-galactosidase under the conditions assayed.

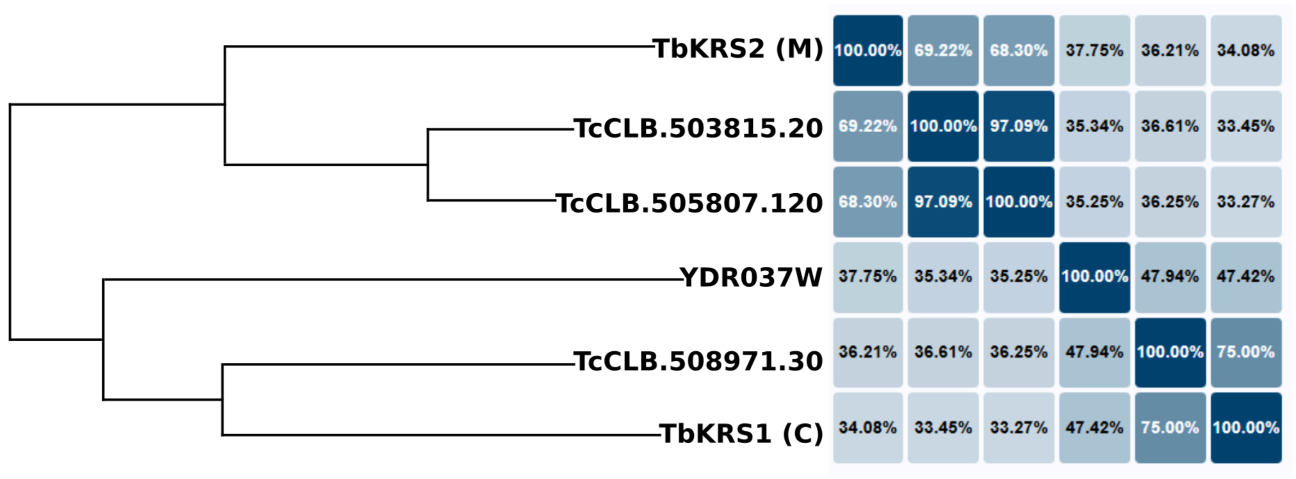

Figure S3. Phylogenetic relationship and sequence similarity of lysyl-tRNA synthetase homologs. The dendrogram shows hierarchical clustering based on pairwise sequence alignment, including *Trypanosoma cruzi* (TcCLB identifiers), *Trypanosoma brucei* cytosolic (TbKRS1-C) and mitochondrial (TbKRS2-M) enzymes, and the *Saccharomyces cerevisiae* ortholog YDR037W. The accompanying heatmap represents pairwise sequence similarity percentages, highlighting the closer relationship of TcCLB.503815.20 and TcCLB.505807.120 to the mitochondrial TbKRS2-M, and TcCLB.508971.30 to the cytosolic TbKRS1-C.

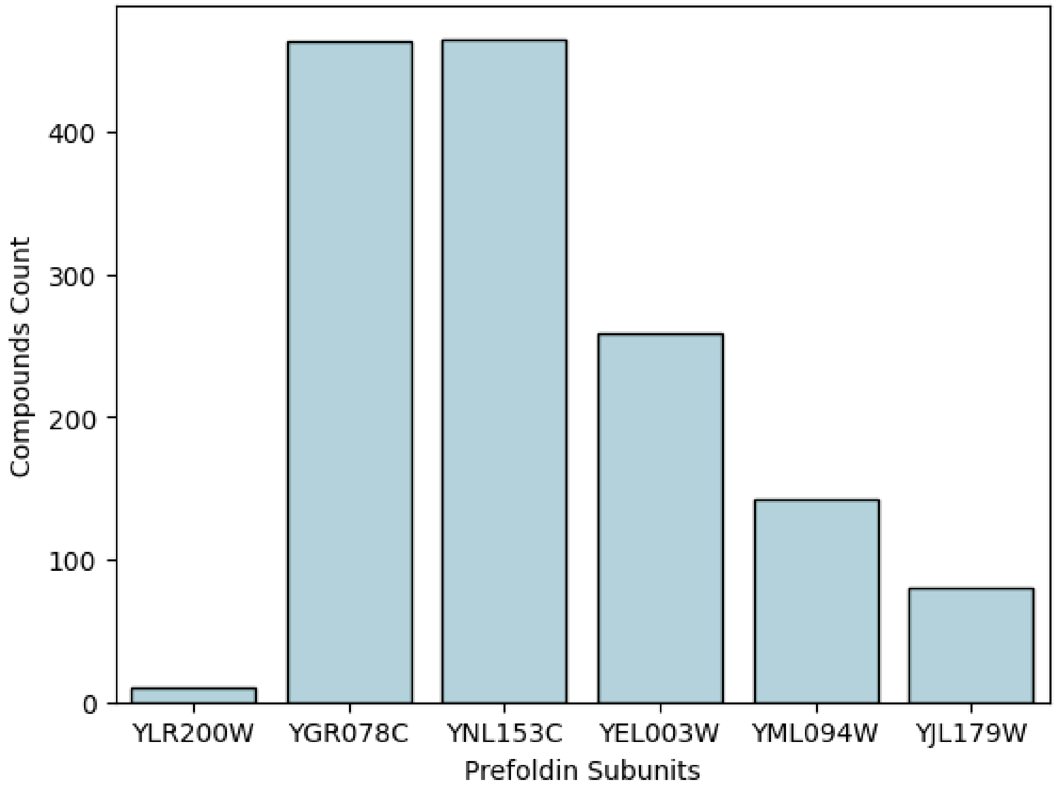

Figure S4. Distribution of compounds through the six subunits of prefoldin. The distribution shows that most prefoldin subunits are associated with a large number of compounds, suggesting prefoldin may behave as a promiscuous target.

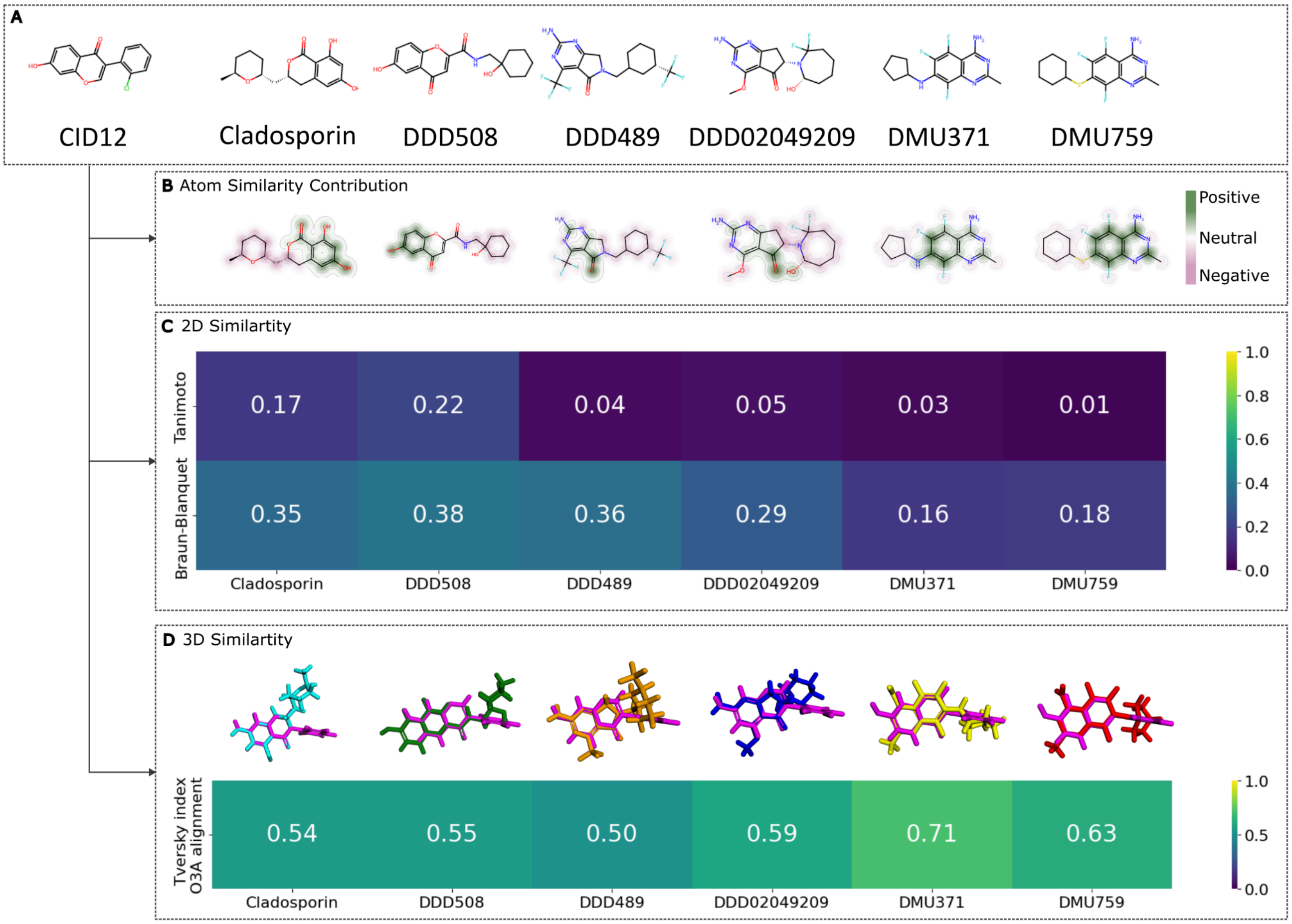

Figure S5. Structure and 2D and 3D similarity analysis of CID12 and other compounds reported in the literature as active against lysyl-tRNA synthetase. Panel A shows the chemical structures of inhibitors reported against lysyl-tRNA synthetase. CID12 was identified as a potential inhibitor in *S. cerevisiae* chemogenomic screens. This target has also been studied in *Cryptosporidium parvum* and *Plasmodium falciparum*, and a few inhibitors have been reported. Panel B shows the atom-level similarity contributions of CID12 compared to each inhibitor, highlighting the substructures that contribute positively to the similarity. Panel C presents the global similarity of CID12 against all reported compounds. Panel D shows the 3D O3A alignment of CID12 and the Tversky index against all reported compounds. 3D comparison can be visualized on an interactive notebook (see Data Availability). Overall, 2D similarity scores were low, but O3A alignment and Tversky scores show partial overlap (shared core with divergent regions), indicating limited structural overlap with known inhibitors.

Supplementary Section 1

Known lysyl-tRNA synthetase inhibitors were compared to CID 12. We conducted pairwise atom-level comparisons using the Similarity Map from RDKit, which highlights how individual molecular substructures contribute to overall similarity. Fingerprint-based similarity was further quantified using the Tanimoto and Braun-Blanquet coefficients (both with a 0–1 range) (1). Three-dimensional similarity was evaluated using O3A (Optimal 3D Alignment) to maximize the overlap of molecular shapes and features. Following alignment, three-dimensional similarity was quantified using the Tversky shape index with α = 0 and β = 1, thereby prioritizing the assessment of how well each target compound could be accommodated within the three-dimensional shape and feature space of the reference molecule (CID12). A summary of these comparisons is presented in Table S5 and Figure S5. Overall, Tanimoto and Braun-Blanquet values were low across these compounds, reflecting only modest shared features at the 2D level. In contrast, Tversky scores were higher, indicating partial structural overlap with known inhibitors. Among these compounds, the *T. cruzi* lysyl-tRNA synthetase inhibitors DMU371 and DMU759, exhibited the highest Tversky scores, suggesting substantial three-dimensional overlap with CID 12 with other minor structural differences.

Table S5. Comparison of known inhibitors of lysyl-tRNA synthetase against CID 12

Compound	Organism	Tanimoto	Braun-Blanquet	Tversky index	Citation
Cladosporin	*P. falciparum*	0.17	0.35	0.54	(2)
DDD508	*C. parvum*	0.22	0.38	0.55	(3)
DDD489	*C. parvum*	0.04	0.36	0.50	(3)
DDD02049209	*M. tuberculosis*	0.05	0.29	0.59	(4)
DMU371	*T. cruzi*	0.03	0.16	0.71	(5)
DMU759	*T. cruzi*	0.01	0.18	0.63	(5)

Recent structural characterization of lysyl-tRNA synthetase across these diverse pathogens, has identified the ATP-binding pocket as a highly conserved and druggable site. These studies have utilized compounds with distinct scaffolds which consistently occupy the adenosine/ATP pocket. Binding is typically stabilized by π-π stacking interactions between the inhibitor's aromatic core, such as the quinazoline ring, with conserved aromatic residues, such as F269 and R481 in *M. tuberculosis* (4) or F317 y R536 in *T. cruzi* (5). Selectivity against the human ortholog was often driven by a few specific residues within the active site; for instance, the presence of V324 and S340 in the parasite pocket versus Q324 and T340 in humans (2). Furthermore, resistance is frequently localized to the hydrophobic ribose pocket, where mutations such as A309L in *Cryptosporidium* (3) or S319L in *T. cruzi* (5) introduce steric clashes that prevent inhibitor binding. Given that CID 12 has a chromen-4-one scaffold and significant 3D similarity to validated inhibitors (most notably the structural overlap with the isocoumarin core of cladosporin and the aromatic rings of the DMU series) we hypothesize that it could behave as an ATP-competitive inhibitor, occupying the adenosine pocket of TcCLB.508971.30 and stabilizing its binding through the same conserved π-π stacking interactions.

References

1. Safizadeh H, Simpkins SW, Nelson J, Li SC, Piotrowski JS, Yoshimura M, Yashiroda Y, Hirano H, Osada H, Yoshida M, Boone C, Myers CL. 2021. Improving Measures of Chemical Structural Similarity Using Machine Learning on Chemical–Genetic Interactions. J Chem Inf Model 61:4156–4172.
2. Hoepfner D, McNamara CW, Lim CS, Studer C, Riedl R, Aust T, McCormack SL, Plouffe DM, Meister S, Schuierer S, Plikat U, Hartmann N, Staedtler F, Cotesta S, Schmitt EK, Petersen F, Supek F, Glynne RJ, Tallarico JA, Porter JA, Fishman MC, Bodenreider C, Diagana TT, Movva NR, Winzeler EA. 2012. Selective and Specific Inhibition of the Plasmodium falciparum Lysyl-tRNA Synthetase by the Fungal Secondary Metabolite Cladosporin. Cell Host Microbe 11:654–663.
3. Caldwell N, Peet C, Miller P, Colon BL, Taylor MG, Cocco M, Dawson A, Lukac I, Teixeira JE, Robinson L, Frame L, Seizova S, Damerow S, Tamaki F, Post J, Riley J, Mutter N, Hanna JC, Ferguson L, Hu X, Tinti M, Forte B, Norcross NR, Campbell PS, Svensen N, Caldwell FC, Jansen C, Postis V, Read KD, Huston CD, Gilbert IH, Baragaña B, Pawlowic MC. 2024. Cryptosporidium lysyl-tRNA synthetase inhibitors define the interplay between solubility and permeability required to achieve efficacy. Sci Transl Med 16:eadm8631.
4. Green SR, Davis SH, Damerow S, Engelhart CA, Mathieson M, Baragaña B, Robinson DA, Tamjar J, Dawson A, Tamaki FK, Buchanan KI, Post J, Dowers K, Shepherd SM, Jansen C, Zuccotto F, Gilbert IH, Epemolu O, Riley J, Stojanovski L, Osuna-Cabello M, Pérez-Herrán E, Rebollo MJ, Guijarro López L, Casado Castro P, Camino I, Kim HC, Bean JM, Nahiyaan N, Rhee KY, Wang Q, Tan VY, Boshoff HIM, Converse PJ, Li S-Y, Chang YS, Fotouhi N, Upton AM, Nuermberger EL, Schnappinger D, Read KD, Encinas L, Bates RH, Wyatt PG, Cleghorn LAT. 2022. Lysyl-tRNA synthetase, a target for urgently needed M. tuberculosis drugs. Nat Commun 13:5992.
5. Tulloch LB, Tawell H, Taylor AE, Lima ML, Dawson A, Carvalho S, Wall RJ, Corpas-Lopez V, Dey G, Duggan J, Magalhaes LG, Torrie LS, Frame L, Robinson D, Patterson S, Tinti M, Weaver GW, Robinson WJ, Cal M, Kaiser M, Mäser P, Sjö P, Perry B, Kelly JM, Francisco AF, Bhambra AS, Wyllie S. 2025. Antitrypanosomal quinazolines targeting lysyl-tRNA synthetase show partial efficacy in a mouse model of acute Chagas disease. Sci Transl Med 17:eadu4564.
